# The Anti-Inflammatory Effects of Formononetin, an Active Constituent of *Pueraria montana* Var. *Lobata*, via Modulation of Macrophage Autophagy and Polarization

**DOI:** 10.3390/molecules30010196

**Published:** 2025-01-06

**Authors:** Linyi Xu, Shuo Zhou, Jing Li, Wenbo Yu, Wenyi Gao, Haoming Luo, Xiaoxue Fang

**Affiliations:** 1 College of Pharmacy, Changchun University of Chinese Medicine, Changchun 130117, China; xulinyi0202@163.com (L.X.); 13364424096@163.com (S.Z.); 18744510205@163.com (J.L.); yuwenbo80@163.com (W.Y.); 2 School of Pharmacy, Changchun University of Chinese Medicine, Changchun 130117, China

**Keywords:** *Pueraria montana* var. *lobata*, formononetin, autophagy, polarization, anti-inflammation

## Abstract

*Pueraria montana* var. *lobata* (Willd.) Maesen & S.M.Almeida ex Sanjappa & Predeep (*P. lobata*) is a medicinal herb widely used in the food and pharmaceutical industries, and studies have shown that *P. lobata* possesses significant anti-inflammatory pharmacological activities. In this paper, a total of 16 compounds were isolated and identified from *P. lobata*, among which compounds **1**–**3**, **7**, **14**, and **16** were isolated from *P. lobata* for the first time. The results of an in vitro anti-inflammatory activity screening assay showed that compounds **1**, **4**, **6**, **8**, and **15** were able to significantly reduce the levels of pro-inflammatory cytokines IL-6 and IL-1β in LPS-induced RAW264.7 macrophages, with the most obvious effect produced by compound **6** (formononetin), while formononetin was able to significantly reduce the number of macrophages at the site of inflammation in transgenic zebrafish. In addition, network pharmacological analysis revealed that the anti-inflammatory activity of formononetin is closely related to autophagy and polarization targets such as *TNF*, *EGFR*, *PTGS2*, and *ESR1*. In vitro validation experiments showed that formononetin could enhance the expression of LCII/LCI and reduce the expression of P62 protein, reduce the expression of CD86, and enhance the expression of CD206, which further indicated that formononetin could reduce inflammation by regulating macrophage autophagy and polarization processes.

## 1. Introduction

Inflammation is a physiological process in which the body triggers a series of immune system interventions in response to injury and infection, allowing various inflammatory factors to interact with each other to synergistically regulate the activation and assembly of immune cells, which effectively removes pathogens and facilitates tissue repair [1]. However, when the inflammatory response is excessive, the release of excessive pro-inflammatory cytokines is closely associated with the development of a wide range of diseases and can induce a range of health problems, including respiratory [2] and neurological [3] systems. Inflammatory diseases have become a major health problem affecting millions of patients worldwide and encompassing multiple disease types. Efforts have been made to delve deeper into the mechanisms underlying the inflammatory response and to actively develop effective anti-inflammatory treatments. However, most of the drugs currently used for the treatment of inflammation are non-steroidal anti-inflammatory drugs (NSAIDs) [4], and their long-term use may trigger side effects such as allergies, or gastrointestinal or central nervous system reactions. Therefore, it is crucial to find natural drugs with low or no toxicity, and herbal medicine has good prospects and applications as a key anti-inflammatory drug.

*Pueraria montana* var. *lobata* (Willd.) Maesen & S.M.Almeida ex Sanjappa & Predeep (*P. lobata*) is a typical medicinal plant, and several studies have shown that it possesses a variety of pharmacological activities, including the improvement of symptoms of diseases such as osteoarthritis [5], mastitis [6], atherosclerosis [7], thyroiditis [8], fatty liver [9], and diabetes mellitus [10], as well as other related diseases. Numerous reports have demonstrated the excellent anti-inflammatory effects of the active components in *P. lobata*. For example, Puerarin, an isoflavonoid compound in *P. lobata*, can effectively inhibit M1-like macrophage activation, thereby ameliorating ulcerative colitis (UC) [11]. *P. lobata* polysaccharides can ameliorate inflammation and lipid peroxidation in mice on alcohol and high-fat diets by restoring intestinal barrier integrity and modulating gut microbiota [12]. In addition, puerarin and puerarin glycosides and their derivatives were able to significantly reduce inflammatory factors and inflammatory markers [13]. In conclusion, *P. lobata* has the potential to be a novel anti-inflammatory agent.

Macrophages have roles in inflammation such as initiating and regulating inflammatory responses, phagocytosis and killing of pathogens, and promoting tissue repair and regeneration. When infection occurs in the organism, macrophages undergo obvious phenotypic and functional changes, polarize to a pro-inflammatory M1 phenotype to respond rapidly and migrate to the site of inflammation, and attract and activate other immune cells to participate in the defense by secreting pro-inflammatory mediators and chemokines. In the later stages of the inflammatory response, macrophages transform into the M2 phenotype with anti-inflammatory and tissue repair functions, releasing anti-inflammatory factors to alleviate the inflammatory response. If pro-inflammatory macrophages persist, they may keep releasing pro-inflammatory factors, leading to the generation of chronic inflammation. In contrast, autophagy can effectively regulate the polarization state of macrophages [14], and autophagy can inhibit the activation of pro-inflammatory signaling pathways, reduce the production and release of pro-inflammatory factors, and then inhibit the pro-inflammatory polarization of macrophages [15]. Meanwhile, autophagy also induces the conversion of macrophages to an anti-inflammatory phenotype, releases anti-inflammatory factors, and participates in tissue repair and inflammation reduction [16]. Liu et al. reportedly found that ubiquitin-specific protease 19 (USP19) was able to inhibit inflammatory responses and promote M2-like macrophage polarization by increasing autophagic flux [17]. Paeoniflorin, an active ingredient of Chinese medicine, inhibits renal inflammation by promoting macrophage polarization from M1 to M2 and modulating KLF4-induced mitochondrial autophagy [18]. These results provide new ideas and potential therapeutic options for the treatment of inflammation-related diseases. Although the anti-inflammatory activity of *P. lobata* has been demonstrated in studies, the pharmacological material basis of *P. lobata*’s anti-inflammatory activity and its mechanism of action against the modulation of macrophage autophagy and polarization have not yet been extensively studied. Therefore, the present study will focus on exploring the anti-inflammatory active components of *P. lobata* and elucidating their specific mechanisms of action.

In this study, we isolated 16 compounds from *P. lobata* and screened the best anti-inflammatory active ingredient as formononetin. The molecular mechanism by which formononetin may treat inflammatory diseases by participating in macrophage autophagy-regulated polarization was analyzed using network pharmacology and verified using in vitro experiments, with a view to laying the foundation for the molecular mechanism by which *P. lobata* exerts its anti-inflammatory pharmacological activity in the future.

## 2. Results

### 2.1. Evaluation of Anti-Inflammatory Activity of Different Extract Layers of P. lobata

**Figure 1:**
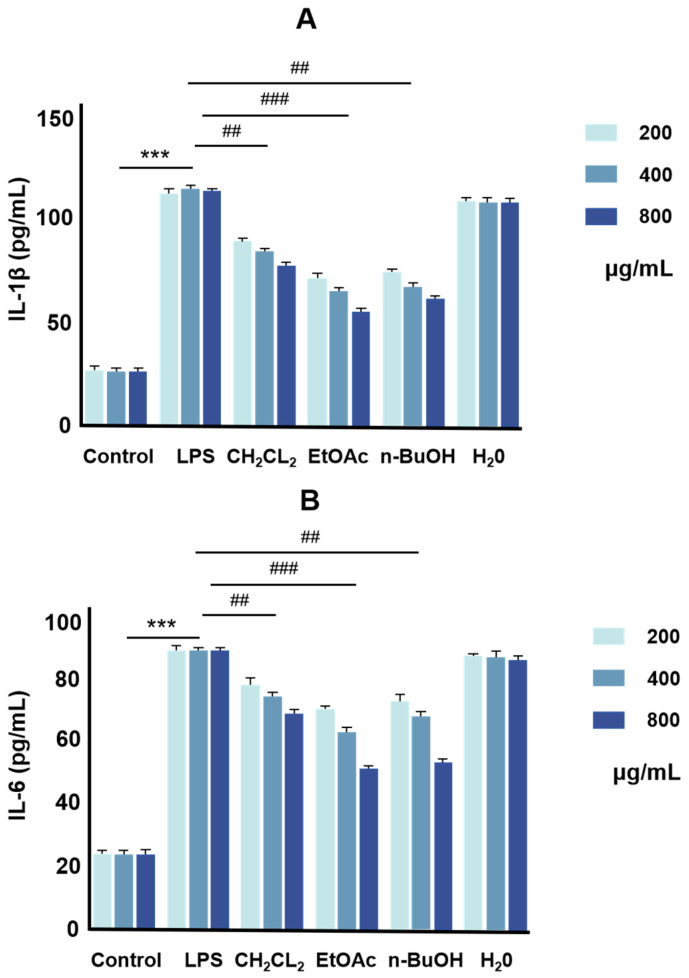
Inflammatory factors (IL-6, IL-1β) content by enzyme-linked immunosorbent assay. (**A**) The inhibitory effect of different extracts of *P. lobata* on IL-1β; (**B**) the inhibitory effect of different extracts of *P. lobata* on IL-6. Values are the mean ± SEM, *n* = 3 (compared to the control group *** *p* < 0.001. Compared to the LPS group, ### *p* < 0.001, ## *p* < 0.01).

The expression of inflammatory factors IL-6 and IL-1β levels in LPS-induced RAW264.7 cells by different layers of P. lobata extracts was detected by ELISA. As shown in Figure 1, the expression levels of IL-1β (Figure 1A) and IL-6 (Figure 1B) were reduced in the cell supernatant after the intervention treatments of the dichloromethane layer [19], ethyl acetate layer [20], and n-butanol layer [21], with the most significant effect produced by the ethyl acetate layer. Therefore, we subsequently isolated and purified the ethyl acetate extracted layer to further determine the material basis of the anti-inflammatory properties of *P. lobata*.

### 2.2. Structural Analysis of Compounds ***1***–***16***

**Figure 2:**
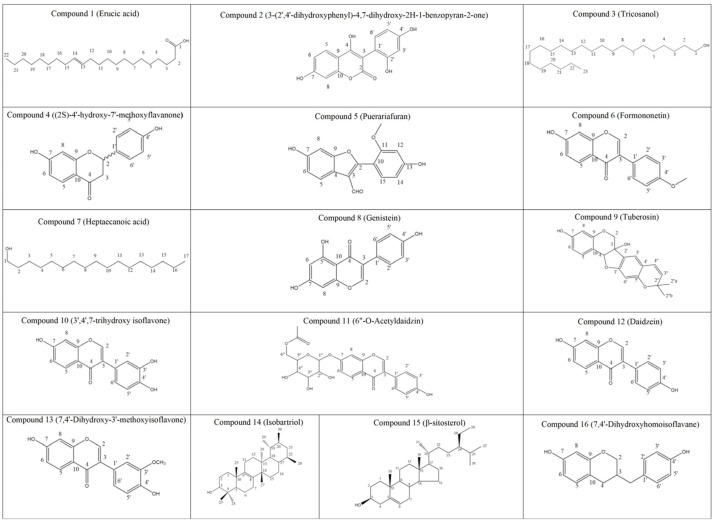
Structure of compounds **1**–**16**.

By comparison with known compounds in the literature [22,23,24,25,26,27,28,29,30,31,32,33,34,35,36,37], the structural formula of compounds **1**–**16** is shown in Figure 2.

### 2.3. Evaluation of Anti-Inflammatory Activity of Compounds ***1***–***16***

**Figure 3:**
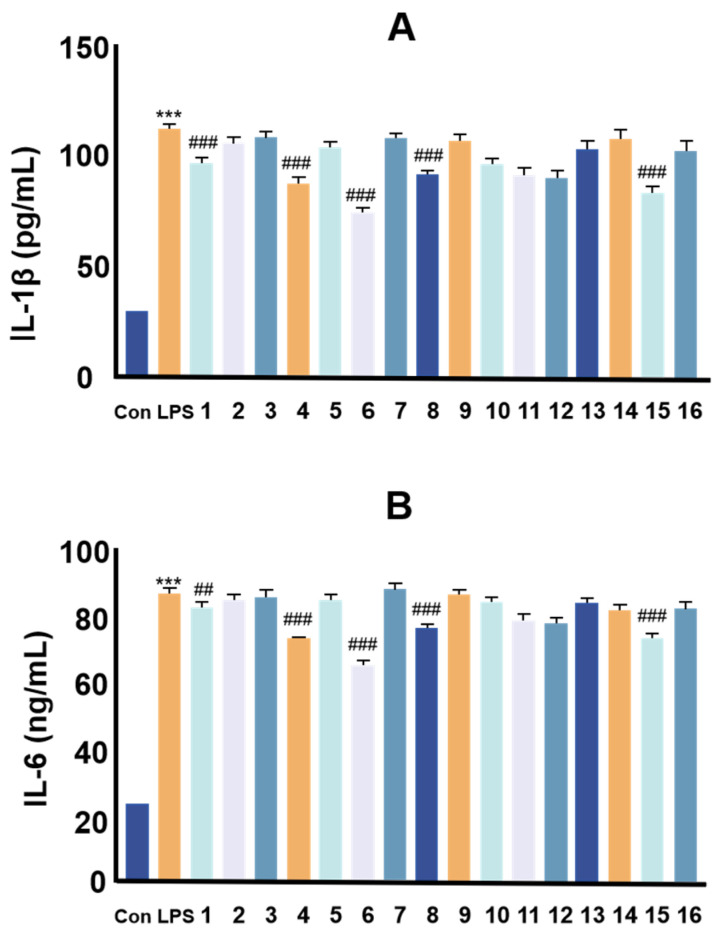
Inflammatory factors (IL-6, IL-1β) content by enzyme-linked immunosorbent assay. (**A**) The inhibitory effect of compound **1**–**16** on IL-1β; (**B**) the inhibitory effect of compound **1**–**16** on IL-6. Values are the mean ± SEM, n = 3 (compared to the control group *** *p* < 0.001. Compared to the LPS group, ### *p* < 0.001, ## *p* < 0.01).

The effect of compounds **1**–**16** in LPS-induced levels of inflammatory factors IL-6 and IL-1β in RAW264.7 cells was examined by ELISA. The results are shown in Figure 3, illustrating that compounds **1**, **4**, **6**, **8**, and **15** exhibited different degrees of anti-inflammatory activities. Among them, compound **6** (formononetin) showed more significant anti-inflammatory effects, suggesting that formononetin is the main anti-inflammatory active component of *P. lobata*.

### 2.4. Effect of Formononetin on RAW 264.7 Cell Viability

**Figure 4:**
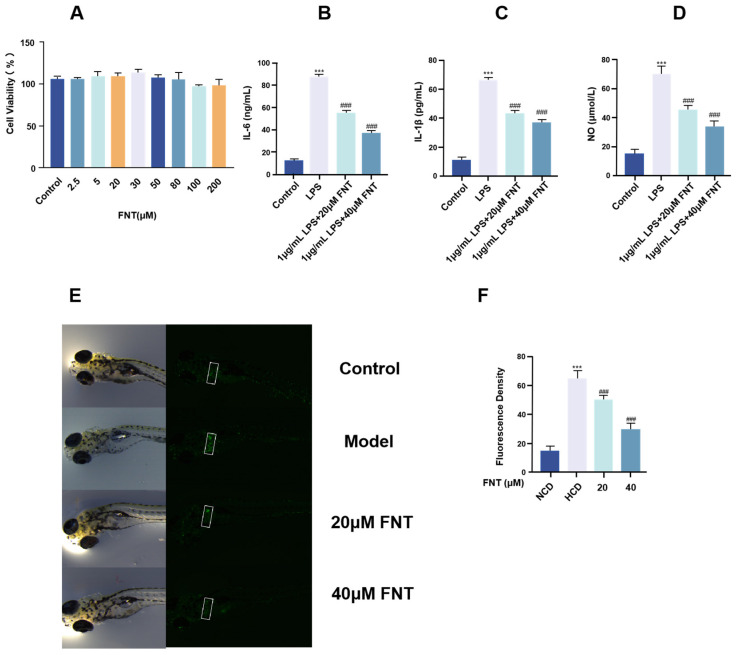
In vitro anti-inflammatory effect of formononetin. (**A**) CCK-8 method for detecting the effect of formononetin on RAW 264.7 cell viability; (**B**–**D**) ELISA method for detecting changes in IL-6, IL-1 β, and NO levels in LPS-induced RAW 264.7 cells after formononetin treatment; values are the mean ± SEM, n = 3 (compared to the control group *** *p* < 0.001. Compared to the LPS group, ### *p* < 0.001); (**E**) fluorescence detection of inflammatory sites in the transgenic zebrafish model of macrophages; (**F**) quantitative analysis of fluorescence intensity of formononetin’s anti-inflammatory effect on the zebrafish inflammatory model. Values are the mean ± SEM, n = 3 (compared to the normal feed (NCD) group *** *p* < 0.001. Compared to the high cholesterol feed (HCD) group, ### *p* < 0.001).

CCK-8 was used to detect the potential toxicity of formononetin on RAW 264.7 cells. As shown in Figure 4A, no significant change in cell viability was observed after 24 h of different concentrations of formononetin on RAW 264.7 cells, indicating that formononetin did not produce cytotoxicity in the concentration range of 2.5–200 μM.

### 2.5. Validation of the In Vitro Anti-Inflammatory Activity of Formononetin

The effects of formononetin on IL-6, IL-1β, and NO levels in LPS-induced inflammation in RAW264.7 cells were determined by ELISA, and the results showed that, compared with the control group, LPS-induced inflammation significantly increased the release of IL-6, IL-1β, and NO from RAW264.7 cells, which were able to be reduced after formononetin treatment. Treatment with formononetin reduced the release of these substances in a dose-dependent manner, and the results are shown in Figure 4B–D.

### 2.6. Validation of In Vivo Anti-Inflammatory Activity of Formononetin

To further confirm the anti-inflammatory effects of formononetin, an inflammation model was induced in macrophage-transgenic zebrafish using a high-cholesterol diet (HCD). The results showed an increased number of macrophages in the liver region of zebrafish in the HCD group, confirming successful model induction. Compared with the HCD group, treatment with formononetin significantly reduced the number of macrophages in the inflamed areas. These findings further support that formononetin exerts anti-inflammatory pharmacological activity by modulating macrophage activity.

### 2.7. Target Screening of Formononetin for the Treatment of Inflammation

**Figure 5:**
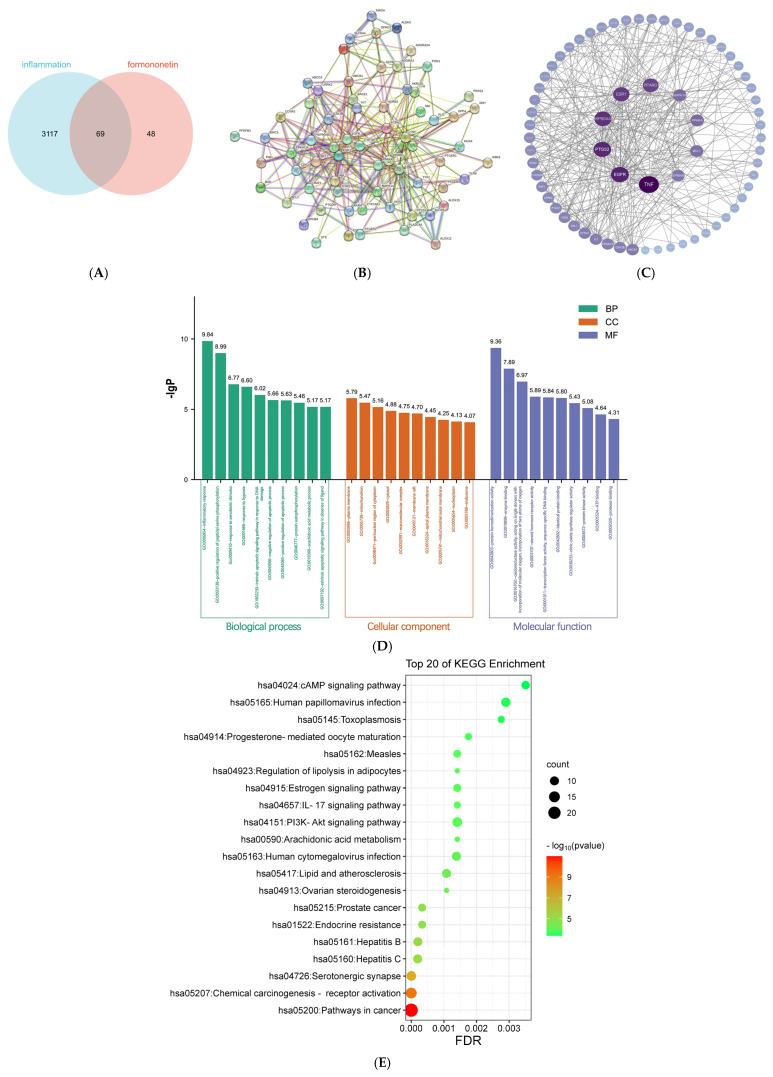
Network pharmacology analysis. (**A**) Venn diagram, showing the common target genes between formononetin and inflammation; (**B**) PPI network analysis, with nodes representing target proteins and edges representing interactions between target proteins; (**C**) key target protein PPI network topology analysis (node size is positively correlated with the degree of purple shadow, with larger values indicating larger nodes and darker purple nodes); (**D**) GO functional enrichment analysis chart; (**E**) KEGG pathway enrichment analysis bubble chart.

Network pharmacology analysis was utilized to elucidate the specific molecular mechanism of action of formononetin in inflammation therapy. A total of 3186 disease targets were firstly obtained by screening using disease-related gene databases (DisGeNET, Genecards, OMIM, TTD, and DrugBank). Meanwhile, 117 drug potential targets were obtained by screening using TCMSP and Swiss Target Prediction databases, and then 69 common targets were obtained by mapping the intersection of disease targets and drug targets, and Venn diagrams were plotted. The results are shown in Figure 5A.

### 2.8. PPI Network Analysis

In order to further identify the core regulatory targets, the 69 common targets obtained above were imported into the STRING database for PPI analysis, and the results are shown in Figure 5B, which consists of 69 target nodes and 350 edges, constituting a complex network of potential inter-target communication. The nodes were then imported into Cytoscape 3.9.0 software for analysis, and ranked according to the degree value, with a higher degree value indicating a stronger correlation between the involvement of these targets in the treatment of inflammation by formononetin, and the results are shown in Figure 5C. Table 1 shows the top 10 targets in terms of degree value, which are *TNF*, *AKT1*, *SRC*, *EGFR*, *CASP3*, *NFKB1*, *JUN*, *MAPK3*, *MMP9*, and *PTGS2*, suggesting that these targets may play a key role in anti-inflammatory treatment of formononetin.

### 2.9. GO Annotation Analysis and KEGG Pathway Enrichment Analysis

In order to elucidate the molecular functions and pathway pathways of the potential anti-inflammatory targets of formononetin, the cross-targets were biologically enriched using the DAVID database, and the results of GO annotation analysis showed that these targets involved 295 biological processes (BPs), which were mainly involved in inflammatory responses and positive regulation of peptide-serine phosphorylation, etc.; 56 cellular components (CCs), mainly plasma membrane and mitochondria, etc.; and 56 molecular functions (MF), mainly protein homodimerization activity and binding enzymes, etc. The top 10 entries of BP, CC, and MF were selected and plotted as bar graphs, as shown in Figure 5D. KEGG enrichment results showed that these targets involved 56 pathways, and the top 20 pathways were selected according to the order of the *p* value to plot the KEGG pathway bubble graphs. The results are shown in Figure 5E, which showed that the targets of formononetin for inflammation were mainly enriched in the pathways of 5-hydroxytryptaminergic synapses, lipids, autophagy, and polarization.

### 2.10. Effect of Formononetin on Autophagy and Polarization Markers in LPS-Stimulated RAW264.7 Cells

**Figure 6:**
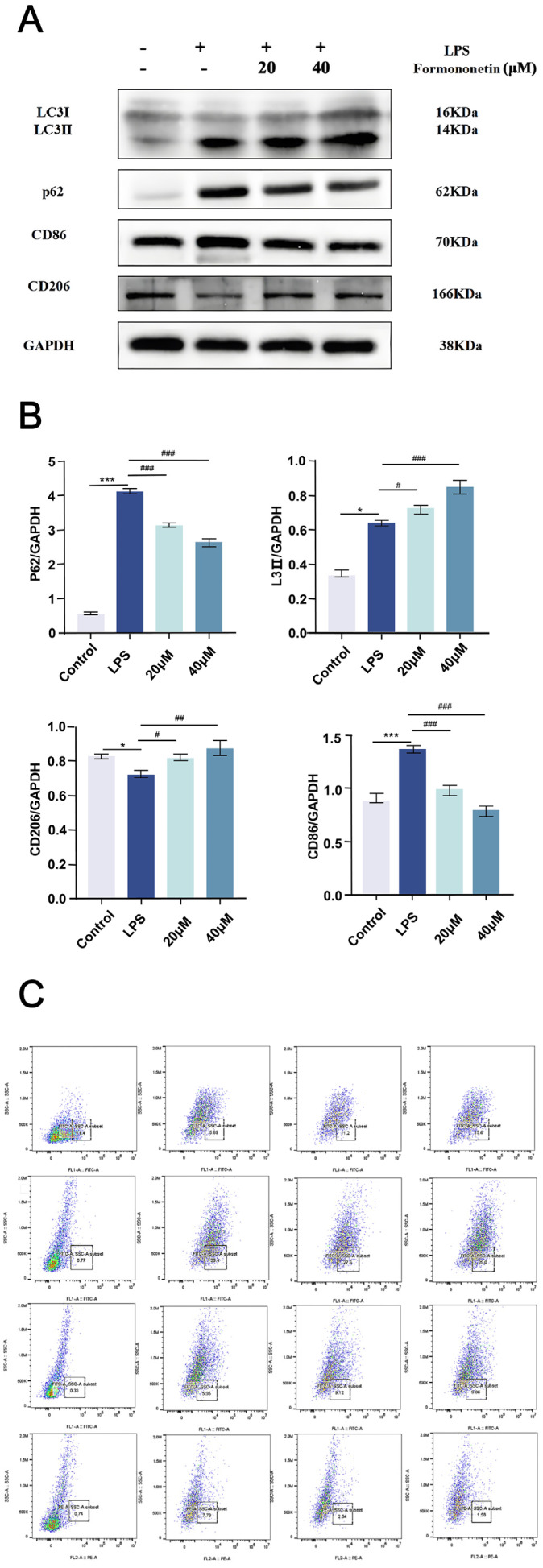
Changes in autophagy and polarized protein levels in LPS-stimulated RAW264.7 cells treated with formononetin. (**A**) Detection of the inhibition or promotion of autophagy and polarization-related proteins in LPS-treated RAW264.7 cells by different concentrations (20 μM, 40 μM) of formononetin; (**B**) quantitative plot; (**C**) flow cytometry. Values are the mean ± SEM, n = 3 (compared to the control group *** *p* < 0.001, * *p* < 0.05. Compared to the LPS group, ### *p* < 0.001, ## *p* < 0.01 or # *p* < 0.05).

Network pharmacological analysis revealed that formononetin may play an anti-inflammatory role by regulating macrophage autophagy and polarization; therefore, in vitro experiments were used for further validation. Firstly, Western blot was used to detect changes in autophagy and polarization protein levels in LPS-stimulated RAW264.7 cells treated with formononetin. As shown in Figure 6A–B, compared with the LPS group, formononetin treatment significantly increased the LCII/I ratio of autophagy marker protein expression and reduced the autophagy substrate protein P62. This suggests that formononetin exerts anti-inflammatory effects by promoting autophagy in macrophages. At the same time, M1 showed a decrease in the expression of macrophage marker protein CD86 and M2 showed an increase in the expression of macrophage marker protein CD206, suggesting that formononetin can promote the transformation of macrophages to M2-type cells. In addition, flow cytometry was used to detect changes in autophagy and polarization protein levels in LPS-stimulated RAW264.7 cells treated with formononetin. As shown in Figure 6C, LC3II/LC3I levels were increased and P62 levels were decreased in formononetin-treated macrophages compared with the LPS group, and the marker CD86+ was decreased in M1 phenotypic macrophages, whereas the marker CD206+ was increased in M2 phenotypic macrophages, and these results were consistent with the analysis of the Western blot results.

## 3. Discussion

Various components of *P. lobata* can act on inflammatory diseases and slow down their progression. Studies have shown that *P. lobata* polysaccharides can reduce joint swelling symptoms, decrease the production of serum inflammatory cytokines TNF-α, IL-1β, and IL-6, and slow down inflammation in osteoarthritis (OA) in rats [5]. A potential role of P. lobata’s active constituents in macrophage inflammatory responses has also been reported in a previous study, where P. lobata exosomes were able to promote M2 macrophage polarization exerting and enhancing anti-inflammatory effects [38]. We found that the potential role of formononetin in macrophage inflammatory responses has been mentioned in previous studies, such as the ability of formononetin to effectively alleviate inflammatory responses such as colitis [39], psoriasiform inflammation [40], and airway inflammation [41].

RAW 264.7 has typical macrophage characteristics, and the cells are able to rapidly activate inflammatory responses after processing specific stimuli, making them important in the study of immune system responses and inflammatory signaling pathways. In our study, formononetin, an active ingredient of *P. lobata*, was able to reduce the levels of pro-inflammatory cytokines IL-6, IL-1β, and NO in vivo and decrease the number of macrophages at the site of inflammation in LPS-stimulated RAW264.7 cell cultures. This suggests that formononetin can reduce the synthesis or release of pro-inflammatory cytokines and exhibit significant anti-inflammatory activity by interfering with the activity of certain cell signaling pathways or transcription factors.

In our study, network pharmacological analyses revealed that the relevant targets and pathways through which formononetin exerts its anti-inflammatory effects are closely related to macrophage autophagy and polarization. For example, TNF targets promote macrophage inflammatory activity by stimulating macrophage-expressed TNF receptor-1 (TNFR1) and TNFR2 [42], whereas TNF exosome-integrated titanium activates macrophage autophagy and promotes M2 macrophage polarization through inhibition of the PI3K/AKT/mTOR pathway under hyperglycemic conditions [43]. Neutrophil extracellular traps (NETs) can activate the EGFR-Beclin-1 signaling pathway and inhibit the formation of autophagosomes in macrophages, thereby promoting the activation of inflammasomes through autophagy, which exacerbates the inflammatory response and contributes to the development of atherosclerosis. [44]. Wang et al. utilized proteomics experiments to analyze the effects of endocytosis of human umbilical cord mesenchymal stem cell (HUC-MSC)-derived small extracellular vesicles (sEVs) to promote EGFR and IGFBP2 binding, leading to downstream STAT3 phosphorylation and IL-10 secretion, and effectively inducing macrophage/microglia phenotypic transition from pro-inflammatory M1 to anti-inflammatory M2 [45]. In addition, the inhibition of EGFR phosphorylation induces the transformation of macrophage M1 phenotype to M2 phenotype to exert anti-inflammatory effects, thereby attenuating the symptoms of acute lung injury triggered by sepsis [46]. In the OA mouse model, Ellagic acid (EA) can selectively inhibit the expression of PTGS2 and the production of prostaglandin E2 (PGE2) in M1 macrophages, thereby suppressing inflammation [47]. In addition, studies have shown that 5-aminolevulinic acid photodynamic therapy (ALA-PDT) can induce M1 polarization of THP-1 macrophages by regulating the PTGS2/PGE2/TLR4/TREM1 axis, thereby exacerbating the inflammatory response, and this is used as a therapeutic approach for chronic inflammatory skin conditions such as acne. [48]. Salmonella-infected macrophages significantly upregulate the expression levels of ESR1 and PIK3C3, genes associated with toxin-induced autophagy, which in turn removes infected cells through increased autophagy [49]. Analysis of the network pharmacology results of the present study indicated that the anti-inflammatory effects exerted by formononetin may be closely related to targets such as *TNF*, *EGFR*, *PTGS2*, and *ESR1*. Therefore, we propose the hypothesis that formononetin may affect macrophage polarization and autophagy by regulating these targets, thus exerting anti-inflammatory effects. However, these hypotheses need to be verified by further experiments, and future studies will focus on validating these potential molecular mechanisms.

## 4. Materials and Methods

### 4.1. Main Instruments and Reagents

The ^1^D-NMR spectra were recorded using a Bruker AV-600 spectrometer 292 (Bruker, Billerica, MA, USA) equipped with a single NMR probe; these spectra were measured in CDCl_3_, CD_3_OD, and C_5_ D_5_N at 400 MHz for ^1^H and 100 MHz for ^13^C. The TLC plate (GF254) and column chromatography silica gel (200–300 mesh) were purchased from Qingdao Marine Chemistry Co., Ltd. (Qingdao, China). The DMEM high-glucose medium (C11995500BT) and fetal bovine serum FBS (30044333) were obtained from Gibco (Oakland, CA, USA). The PBS (SH30256), trypsin (SH30042), and penicillin–streptomycin double antibiotic (SV30010) were from Hyclone (Logan, UT, USA). The LPS (L4391) was from SIGMAG, while the CCK-8 kit (IV 08-500) was from Invigentech (Irvine, CA, USA).

### 4.2. Sources of Medicinal Herbs

The P. lobata was purchased from Beiyao Pharmaceutical Group Co., Ltd. (Changchun, China), in May 2023 and authenticated by Lili Weng, a professor at Changchun University of Traditional Chinese Medicine.

### 4.3. Source of Cells

RAW 264.7 is a macrophage cell line that was established from a tumor in a male mouse induced with the Abelson murine leukemia virus. The source information for cell lines is referenced in an article on the ATCC Cell Bank (https://www.atcc.org/cell-products, accessed on 8 June 2023).

### 4.4. Extraction and Extraction of the Chemical Constituents of P. lobata

A total of 10 kg of *P. lobata* herb was weighed, and 34 L of 70% ethanol was added before reflux extraction was undertaken three times, each time for 7 days, then filtered using a 200 mesh filter cloth, and the resulting extract was concentrated under reduced pressure using a rotary evaporator to remove ethanol to obtain the *P. lobata* crude extract (4.9 kg, yield 49.0%). The crude extract was dissolved in 1.2 times the amount of distilled water, and liquid–liquid extraction was carried out using dichloromethane, ethyl acetate, and n-butanol, respectively, three times, and then concentrated under reduced pressure to obtain the extracts of the extracts of each layer (the weights of the extracts of the dichloromethane layer, the ethyl acetate layer, and the n-butanol layer were 69.1 g, 108.8 g, and 1984.9 g, respectively).

### 4.5. Screening of Anti-Inflammatory Activity of Different Extracts of P. lobata Extracts

RAW264.7 cells were seeded in 60 mm cell culture dishes (4.5 × 10⁵ cells/dish) and incubated overnight. Different *P. lobata* extracts were added, and the cultures were incubated at 37 °C for 1 h. Subsequently, 1 μg/mL LPS was added, and the cells were incubated for another 24 h at 37 °C. The levels of IL-6 and IL-1β in the cell supernatants were then measured using ELISA (Jiangsu Meimian Industrial Co., Ltd., Yancheng, China).

### 4.6. Isolation and Purification of Chemical Constituents of P. lobata Ethyl Acetate Layer Extracts

The ethyl acetate layer extract (105 g) was taken and dissolved using dichloromethane and methanol, then silica gel was added (extract: silica gel = 1:1, M/M), dried under reduced pressure, and eluted with a gradient of petroleum ether–ethyl acetate and dichloromethane–methanol (petroleum ether: ethyl acetate = 8:1 → 4:1; dichloromethane: methanol = 20:1 → 5:1), and the elution fractions were combined by thin-layer chromatographic detection.

Fr.E-2 (0.6733 g) underwent further separation by a normal-phase silica gel column (3 × 47 cm) with gradient elution of petroleum ether–ethyl acetate (petroleum ether: ethyl acetate = 35:1 → 30:1 → 25:1 → 20:1 → 15:1 → 10:1 → 4:1 → 2:1, *v*/*v*), and the obtained fractions of each elution stream were detected using thin-layer chromatography and then combined. Fr.E-2-5 (0.4024 g) underwent further separation by a reverse silica gel column (2.5 × 40 cm), the separation was carried out by gradient elution with methanol–purified water and methanol–acetone (methanol: purified water = 7:3 → 9:1 → methanol: acetone = 7:3, *v*/*v*), and the obtained fractions of each elution stream were combined after their detection using thin-layer chromatography.

Fr.E-3 (0.5109 g) was separated on a normal-phase silica gel column (2.5 × 47 cm), the separation was continued by gradient elution with petroleum ether–ethyl acetate (petroleum ether: ethyl acetate = 35:1 → 30:1 → 25:1 → 20:1 → 15:1 → 10:1 → 4:1 → 2:1, *v*/*v*), and the eluted fractions were combined after detection by thin-layer chromatography. Fr.E-3-9 (0.5011 g) was further separated on a reverse silica gel column (2.5 × 36 cm) with a gradient elution of methanol–purified water (methanol: purified water = 5.5:4.5 → 7:3 → 9:1, *v*/*v*), and the resulting elution fractions were detected by thin-layer chromatography and combined.

Fr.E-6 (0.9079 g) was separated on a normal-phase silica gel column (3 × 50 cm), the separation was continued by gradient elution with petroleum ether–ethyl acetate (petroleum ether: ethyl acetate = 10:1 → 7:1 → 5:1 → 2:1, *v*/*v*), and the obtained fractions of each elution stream were detected by thin-layer chromatography and then combined. Fr.E-6-10 (0.2517 g) was separated on a reversed-phase silica gel column (2.5 × 45 cm). E-6-10 (0.2517 g) was separated on a reversed silica gel column (2.5 × 45 cm), the separation was continued with a gradient elution of methanol–purified water (methanol: purified water = 4:6 → 4.5:5.5 → 5.5:4.5 → 7.5:2.5 → 8.5:1.5, *v*/*v*), and the resulting fractions of each eluent were detected by thin-layer chromatography and then combined.

Fr.E-7 (14.4289 g) underwent further separation by a silica gel column (5 × 40 cm) with gradient elution by petroleum ether–ethyl acetate and dichloromethane–methanol (petroleum ether: ethyl acetate = 8:1 → 6:1 → 3:1; dichloromethane: methanol = 40:1, *v*/*v*), and the obtained fractions of each elution stream were combined after their detection using thin-layer chromatography. Fr.E-7-4 (0.2019 g) underwent further separation by a reverse silica gel column (2.5 × 40 cm) with a gradient elution of methanol–purified water (methanol: purified water = 4:6 → 5.5:4.5 → 7:3, *v*/*v*), and the obtained fractions of each elution stream were combined after their detection using thin-layer chromatography. Fr.E-7-5 (0.1903 g) was separated on a reversed-phase silica gel column (2.5 × 40 cm), and the separation was continued by gradient elution with methanol-purified water (methanol: purified water = 4:6→4.5:5.5→5.5:4.5, *v*/*v*), and the obtained fractions of the respective elution streams were detected and combined using thin-layer chromatography. With E-7-5-6 (0.0153 g), the separation was continued by a reversed-phase silica gel column (1.2 × 60 cm) with a gradient elution of methanol–purified water (methanol: purified water = 5.2:4.8 → 5.7:4.3 → 6.5:3.5, *v*/*v*), and the obtained fractions of each eluted stream were detected and combined by thin-layer chromatography. With Fr.E-7-5-6 (0.0153 g), the separation was continued by a reversed-phase silica gel column (1.2 × 60 cm) with a gradient elution of methanol–purified water (methanol: purified water). E-7-6 (0.6419 g) was separated on a reversed-phase silica gel column (2.5 × 35 cm). The separation was continued by gradient elution with methanol–purified water (methanol: purified water = 5.5:4.5 → 7:3, *v*/*v*), and the eluted fractions were combined by thin-layer chromatography.) E-7-6-3 (0.1969 g) was separated on a silica gel column (2.5 × 50 cm) with a gradient elution of petroleum ether–ethyl acetate–methanol (petroleum ether: ethyl acetate: methanol = 40:1:1 → 35:1:1 → 30:1:1, *v*/*v*), and the eluted fractions were then analyzed by thin-layer chromatography and combined. E-7-6-3-5 (0.143 g) underwent further separation by a reversed-phase silica gel column (2.5 × 45 cm). The separation was carried out by gradient elution with methanol–purified water (methanol: purified water = 6:4 → 8:2, *v*/*v*), and the obtained fractions of each elution stream were detected using thin-layer chromatography and then combined. Fr. 4 (0.1391 g) underwent further separation by a normal-phase silica gel column (2 × 50 cm) with a gradient elution of petroleum ether–ethyl acetate–methanol (petroleum ether: ethyl acetate: methanol = 40:1:1 → 35:1:1 → 30:1:1, *v*/*v*), and the elution streams obtained were combined after their detection using thin-layer chromatography. Fr.E-7-6-4-4 (0.0698 g) was passed through a reversed-phase silica gel column (2.5 × 45 cm) to continue the separation, using a gradient elution with methanol–purified water (methanol: purified water = 5.7:4.3, *v*/*v*), and the obtained fractions of each elution stream were combined after their detection using thin-layer chromatography. Fr.E-7-6-4-4-2 (0.0577 g) was passed through a reversed-phase silica gel column (2.5 × 45 cm). The separation was continued with a gradient elution of methanol–purified water (methanol: purified water = 4.5:5.5 → 5.1:4.9, *v/v*), and the obtained fractions of each elution stream were detected using thin-layer chromatography and combined. Fr.E-7-7 (3.6688 g) underwent further separation by a reversed-phase silica gel column (3.5 × 50 cm), and was eluted by a gradient elution of methanol–purified water (methanol: purified water = 4.5:5:1 → 7:3, *v*/*v*). The obtained fractions of each elution stream were detected by thin-layer chromatography and combined. Fr.E-7-7-3 (0.3570 g) underwent continued purification and was separated by a normal-phase silica gel column. E-7-7-3 (0.3570 g) underwent continued purification and was separated by a normal-phase silica gel column (3 × 50 cm), and then eluted with a gradient of dichloromethane–methanol (dichloromethane: methanol = 35:1 → 25:1 → 20:1 → 15:1 → 10:1 → 7:1 → 4:1, *v*/*v*), and the obtained fractions of each elution stream were detected by using thin-layer chromatography and then combined. E-7-7-5 (0.3198 g) underwent continued purification and was isolated by passing through a normal-phase silica gel column (2.5 × 50 cm), and then the obtained fractions were combined after detection using thin-layer chromatography. E-7-7-5 (0.3198 g) was separated by a normal-phase silica gel column (2.5 × 50 cm) and eluted with a gradient of dichloromethane–methanol (dichloromethane: methanol = 30:1 → 25:1 → 20:1 → 15:1 → 10:1 → 5:1, *v*/*v*), and the eluted fractions were detected by thin-layer chromatography and then combined. E-7-7-6 (0.8028 g) was separated by a normal-phase silica gel column (3.5 × 50 cm) and detected by thin-layer chromatography. E-7-7-6 (0.8028 g) underwent continued separation by a normal-phase silica gel column (3.5 × 50 cm) with a gradient elution of dichloromethane–methanol (dichloromethane: methanol = 40:1 → 25:1 → 20:1 → 10:1, *v*/*v*), and the obtained fractions of each elution stream were combined after their detection using thin-layer chromatography. Fr.E-7-7-9 (0.3959 g) underwent continued separation by a reversed-phase silica gel column (2.5 × 45 cm) with a gradient elution of methanol–purified water (methanol: methanol: water) and was then combined after detection. E-7-9-5 (0.0565 g) was separated on a normal-phase silica gel column (1.5 × 50 cm), and the separation was continued on a dichloromethane–ethyl acetate gradient elution (dichloromethane: ethyl acetate = 22:1 → 12:1, *v*/*v*), and the obtained fractions of each elution stream were combined after their detection using thin-layer chromatography.

Fr.E-8 (13.9348 g) underwent continued separation by a normal-phase silica gel column (10 × 40 cm) with a gradient elution of dichloromethane–methanol (dichloromethane: methanol = 35:1 → 35:1 → 20:1 → 10:1, *v*/*v*), and the obtained fractions of each elution stream were combined after their detection using thin-layer chromatography. Fr.E-8-4 (0.1255 g) underwent continued separation by a reversed-phase silica gel column (2.5 × 50 cm), the separation was carried out by gradient elution with methanol–purified water (methanol: purified water = 4.9:5.1 → 5.2:4.8 → 5.8:4.2 → 7:3 → *v*/*v*), and the obtained fractions of each elution stream were combined after their detection using thin-layer chromatography. Fr.E-8-9 (4.2388 g) underwent continued purification and was separated by a reversed-phase silica gel column (3.5×50 cm), and the gradient elution was performed with methanol-purified water (methanol: purified water=4.5:5.5→7.5:2.5, *v*/*v*), and the obtained fractions of each elution stream were detected and combined after the detection of each stream using thin-layer chromatography. Fr.E-8-9-1 (1.4190 g) underwent continued separation by a normal-phase silica gel column (3 × 57 cm), and was combined by gradient elution with methylene chloride–methanol (methylene chloride–methanol). E-8-9-3 (1.3106 g) was separated on a reversed-phase silica gel column (3.5 × 58 cm), and the separation was continued on a gradient elution with dichloromethane–methanol (dichloromethane–methanol = 40:1 → 20:1 → 20:1, *v*/*v*).

### 4.7. Cell Culture

RAW264.7 cells were cultured in a DMEM culture medium containing 10% fetal bovine serum and 1% penicillin–streptomycin double antibiotic at 37 °C in a 5% CO_2_ incubator.

### 4.8. Cell Viability Assay

The effect of compound formononetin on the viability of RAW264.7 cells was determined by a CCK-8 assay. RAW264.7 cells were inoculated in 96-well plates at 1.5 × 10^4^ per well and incubated at 37 °C in a 5% CO_2_ incubator for 24 h. Subsequently, different concentrations of formononetin (2.5 μM, 5 μM, 20 μM, 30 μM, 50 μM, 80 μM, 100 μM, and 200 μM) were added to the cells and incubated for an additional 24 h. After incubation, 10 μL of CCK-8 reagent was added to each well, and the cells were further incubated at 37 °C for 30 min in the dark. The absorbance at 450 nm was measured using the SpectraMax^®^ Paradigm^®^ Multi-Mode Detection Platform (SER33270-1236, Molecular Devices, San Jose, CA, USA) to evaluate cell viability.

### 4.9. Screening of Compounds ***1***–***16*** for Anti-Inflammatory Activity

RAW264.7 cells were seeded in 60 mm cell culture dishes (4.5 × 10⁵ cells/dish) and incubated overnight. Compounds **1**–**16** were added, and the cultures were incubated at 37 °C for 1 h. Subsequently, 1 μg/mL LPS was added, and the cells were incubated for another 24 h at 37 °C. The levels of IL-6 and IL-1β in the cell supernatants were then measured using ELISA.

### 4.10. In Vitro Anti-Inflammatory Activity of Formononetin

RAW264.7 cells were seeded in 60 mm cell culture dishes (4.5 × 10^5^ cells/dish) and incubated overnight. Formononetin was added, and the cultures were incubated at 37 °C for 1 h. Subsequently, 1 μg/mL LPS was added, and the cells were incubated for another 24 h at 37 °C. Cell supernatants were collected. NO levels were determined using a nitric oxide colorimetric assay kit (Nanjing Jiancheng Bioengineering Research Institute, Nanjing, China), an IL-6 ELISA kit and an IL-1β ELISA kit (Jiangsu Meimian Industrial Co., Ltd., Yancheng, China) were used to determine IL-6 and IL-1β.

### 4.11. In Vivo Anti-Inflammatory Activity Assay of Formononetin

The transgenic zebrafish model CZ98 (ihb20Tg, Tg(mpeg1:EGFP)) was purchased from the Institute of Hydrobiology, Chinese Academy of Sciences [50]. Transgenic zebrafish embryos were incubated until 5 days post-fertilization (dpf), and healthy larvae were selected and transferred into 6-well plates for cultivation. The larvae were divided into four groups: normal chow diet (NCD) group, high-cholesterol diet (HCD) group, HCD + 20 μM magnolol group, and HCD + 40 μM magnolol group, with 20 larvae in each group. The NCD group was fed with normal chow, while the HCD group was fed with a 10% high-cholesterol diet (for the high-cholesterol diet, cholesterol was dissolved in anhydrous ethanol and mixed with normal chow, then the ethanol was evaporated and the mixture ground). Each group of zebrafish was fed twice daily at scheduled times with 5 mg of feed per well per feeding. Each feeding lasted 30 min, and the feeding regimen continued for 8 days. The migration of macrophages to the sites of inflammation was observed in each group [51].

### 4.12. Network Pharmacology

The TCMSP (http://old.tcmsp-e.com/tcmsp.php, accessed on 12 October 2024) and Swiss Target Prediction database (http://www.SwissTargetPrediction.ch, accessed on 12 October 2024) were used to collect the “formononetin” component-related targets [52,53]. Meanwhile, the disease databases OMIM (http://omim.org/, accessed on 12 October 2024), GeneCards (https://www.genecards.org/, accessed on 12 October 2024), and DisGeNET (https://disgenet.com/, accessed on 12 October 2024) were used to collect the targets related to the components of “formononetin” by using “inflammation” as the search term, TTD (https://db.idrblab.net/ttd/, accessed on 12 October 2024), and DrugBank (https://go.drugbank.com/, accessed on 12 October 2024) were used to collect potential disease targets from the merged databases. The Uniprot database was accessed, the gene names and UniProt IDs of the targets were checked and corrected, and duplicates were removed to obtain the disease targets and used for the next step of the study [54,55,56,57,58]. Protein–protein interaction (PPI) networks of cross-targets were constructed using the STRING online database. The species was set as “*Homo sapiens*”, and the key targets with confidence >0.400 were imported into the database, the nodes with confidence scores >0.95 were searched, and the free nodes were hidden. The remaining results were saved in TSV format [59]. These TSV files were then imported into Cytoscape 3.9.0 software to visualize the PPI network. Network topology analysis was performed using the network analyzer function in Cytoscape 3.9.0 software, key targets were sorted according to the degree of freedom values, and the top 10 targets with the highest degree values were selected as core targets for GO and KEGG analysis.

### 4.13. Western Blotting

Cells were washed with PBS, centrifuged, and the cell precipitate was collected. Protein extraction was performed using RIPA (R0010, Solarbio, Beijing, China), with protease inhibitors added proportionally. After sonication, the samples were heated at 95 °C for 10 min. The protein concentration in the supernatant was measured using an Ultra-micro spectrophotometer (Aurora-900, Hangzhou Haipui Instrument Co., Ltd., Hangzhou, China). Protein electrophoresis was conducted using SDS-PAGE (P1200, Solarbio, Beijing, China), and the target protein bands were transferred to a PVDF membrane. The membranes were blocked with 5% skim milk and then incubated overnight at 4 °C with specific primary antibodies. The primary antibodies used were CD86 Polyclonal antibody (No. 13395-1-AP, Proteintech, Chicago, IL, USA), CD206 Polyclonal antibody (No. 18704-1-AP, Proteintech, Chicago, IL, USA), P62/SQSTM1 Polyclonal antibody (No. 18420-1-AP, Proteintech, Chicago, IL, USA), and LC3 Recombinant antibody (No. 81004-1-RR, Proteintech, Chicago, IL, USA). To standardize the protein loading, GAPDH Monoclonal antibody (No. 60004-1-Ig, Proteintech, Chicago, IL, USA) was used as the loading control. Subsequently, the membranes were incubated with HRP-conjugated secondary antibodies that bind to the primary antibodies, such as HRP-conjugated Goat Anti-Rabbit IgG (H + L) (No. SA00001-2, Proteintech, Chicago, IL, USA) or HRP-conjugated Goat Anti-Mouse IgG (H + L) (No. SA00001-1, Proteintech, Chicago, IL, USA), at room temperature for 1 h. Finally, the target proteins were visualized using the iBright^TM^ FL1000 Imaging System (A32752, Thermo Fisher Scientific, Waltham, MA, USA).

### 4.14. Flow Cytometry

RAW264.7 cells were seeded in 60 mm cell culture dishes (4.5 × 10^5^ cells/dish) and incubated overnight. Formononetin was added, and the cultures were incubated at 37 °C for 1 h. Subsequently, 1 μg/mL LPS was added, and the cells were incubated for another 24 h at 37 °C. Macrophage single cell suspensions were collected into sterile EP tubes. The cells were centrifuged at 800× *g* for 5 min, and the supernatant was removed. The sample was resuspended in cold 500 μLPBS and 0.5 μL of primary antibody was added and incubated in an ice-water bath for 30 min, protected from light. At the end of the incubation, the sample was centrifuged and the supernatant was discarded. The sample was resuspended in cold 500 μLPBS and 1 μL of fluorescently labeled secondary antibody was added and incubated in an ice-water bath for 30 min, protected from light. The sample was then centrifuged and washed twice with PBS to remove excess antibody. Finally, it was filtered through a mesh filter (200 mesh) and analyzed by Beckman Coulter CytoFLEX Model A00-1-1102 (Brea, CA, USA).

### 4.15. Analysis of Data

Data were evaluated using multiple comparison tests and one-way analysis of variance (ANOVA). Differences were considered highly significant when *p* < 0.01. All results are expressed as mean ± SD, n = 3. Graph Pad Prism 9.5 was used for experimental graphical data processing.

## 5. Conclusions

In this study, 16 compounds were isolated from *P. lobata*, among which Erucic Acid, 3-(2′,4′-Dihydroxyphenyl)-4,7-dihydroxy-2H-1-benzopyran-2-one, Tricosanol, Heptaecanoic Acid, Isobartriol, and 7,4′-Dihydroxyhomoisoflavane were isolated from *P. lobata* for the first time. Then, among the 16 compounds screened, formononetin exhibited the most significant anti-inflammatory activity. In vitro experiments showed that formononetin was able to reduce the levels of IL-6, IL-1β, and NO produced by LPS-stimulated RAW264.7 cells. Network pharmacological analysis revealed that formononetin could exert anti-inflammatory effects by regulating macrophage polarization and autophagy through the targets of *TNF*, *EGFR*, *PTGS2*, and *ESR1*, and in vitro experiments further verified that formononetin increased the expression of autophagy markers LC3II and M2 macrophage marker CD206, and decreased the expression of autophagy marker p62 and M1 macrophage marker CD86. This study provides a direction for the subsequent research on the anti-inflammatory activity and molecular mechanism of formononetin.

## Figures and Tables

**Table 1:** Key anti-inflammatory targets of formononetin.

Number	Gene Name	Degree	Betweenness	Closeness
1	*TNF*	40	862.8772	0.70
2	*EGFR*	33	536.09656	0.66
3	*PTGS2*	32	544.1516	0.65
4	*HSP90AA1*	30	267.754	0.62
5	*ESR1*	28	414.8014	0.63
6	*PPARG*	26	233.52591	0.61
7	*PPARA*	18	109.59851	0.55
8	*MCL1*	17	99.20689	0.54
9	*ABCB1*	15	135.8349	0.54
10	*PRKACA*	14	209.27641	0.50

## Data Availability

The original contributions presented in the study are included in the article (and Supplementary Materials), further inquiries can be directed to the corresponding authors.

## Supplementary Materials

The following supporting information can be downloaded at: https://www.mdpi.com/article/10.3390/molecules30010196/s1, Figures S1–S35: NMR and MS spectra of compounds **1**–**16**, Figure S36: Structure analysis of compounds **1**–**16**, Table S1: Anti-inflammatory targets of formononetin; Table S2: Intersecting target protein interactions; Table S3: GO annotation enrichment analysis; Table S4: KEGG pathway enrichment analysis [22,23,24,25,26,27,28,29,30,31,32,33,34,35,36,37].
