# Supplementary material for: The Anti-Inflammatory Effects of Formononetin, an Active Constituent of Pueraria montana Var. Lobata, via Modulation of Macrophage Autophagy and Polarization
Source: Molecules. 2025 Jan 6;30(1):196. doi: 10.3390/molecules30010196 (PMC11721999; doi:10.3390/molecules30010196)
Supplement: Supplementary file 1 [file molecules-30-00196-s001.zip › molecules-3370804-supplementary.pdf]

Supplementary Materials

The Anti-Inflammatory Effects of Formononetin, an Active Constituent of *Pueraria montana* Var. *Lobata*, via Modulation of Macrophage Autophagy and Polarization

Linyi Xu [1,2], Shuo Zhou [1,2], Jing Li[1,2] , Wenbo Yu [1,2], Wenyi Gao [1,2,*], Haoming Luo [1,2,*]and Xiaoxue Fang [1,2,*]

[1] Changchun University of Chinese Medicine, Changchun 130117, China
[2] School of Pharmacy, Changchun University of Chinese Medicine, Changchun 130117, China
* Correspondence: gaowy@ccucm.edu.cn (W.G.); luo.haoming@163.com (H.L.); fangxiaoxue1996@163.com (X.F.)

Table of Contents

1. NMR and MS spectra of compounds 1-16.. - 1 -

2. Structure analysis of compounds 1-16...- 17 -

3. Anti-inflammatory targets of formononetin.. - 21 -

4. Intersecting target protein interactions.. - 22 -

5. GO annotation enrichment analysis.. - 23 -

6. KEGG pathway enrichment analysis.. - 24 -

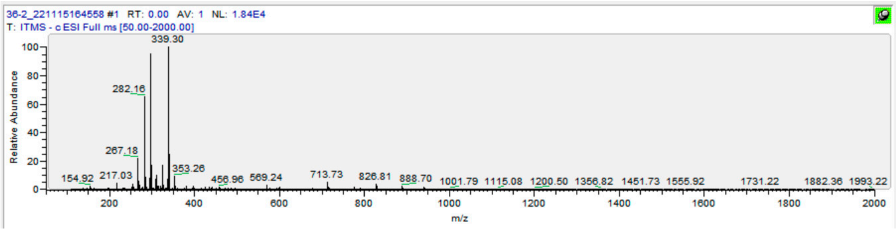

Figure S1. Mass spectra of Coumpound 1

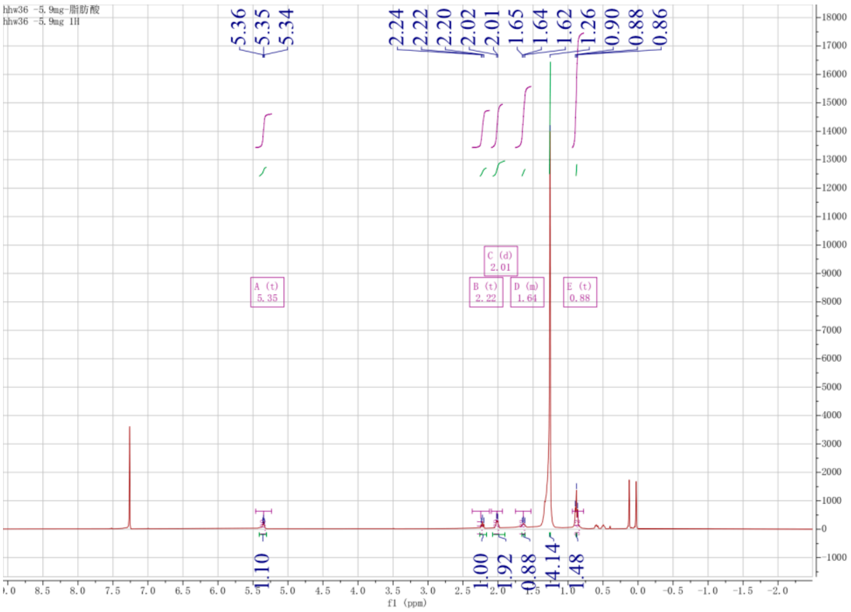

Figure S2. Coumpound1 ^{1}H-NMR

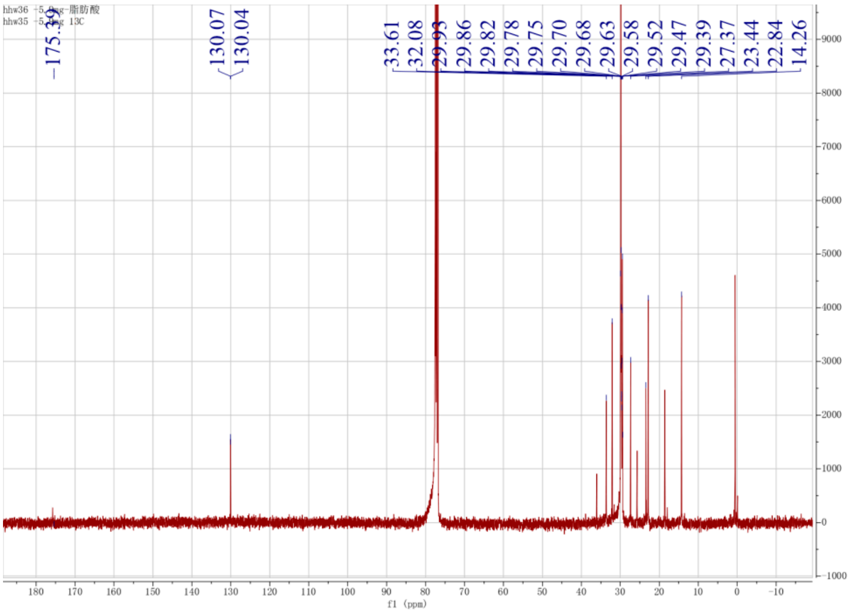

Figure S3. Coumpound1 ^{13}C-NMR

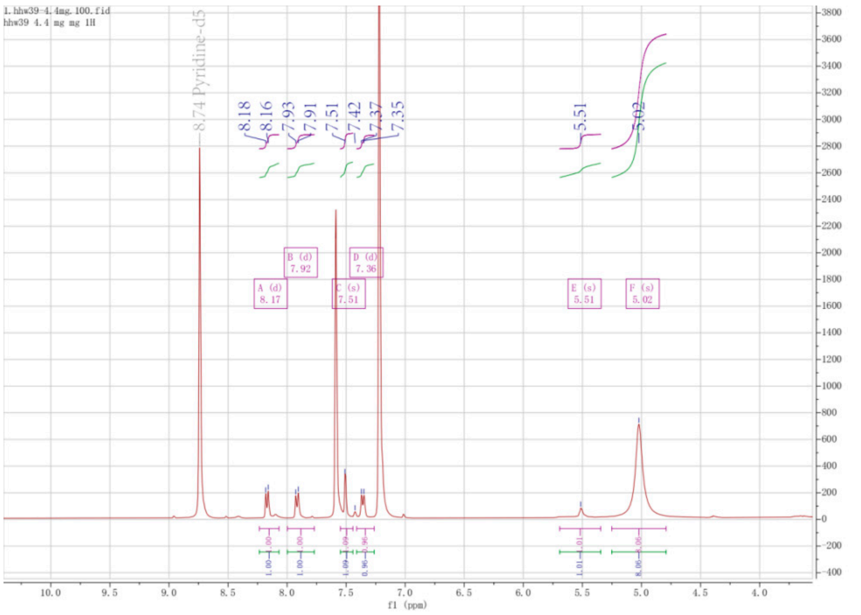

Figure S4. Coumpound 2 ^{1}H-NMR

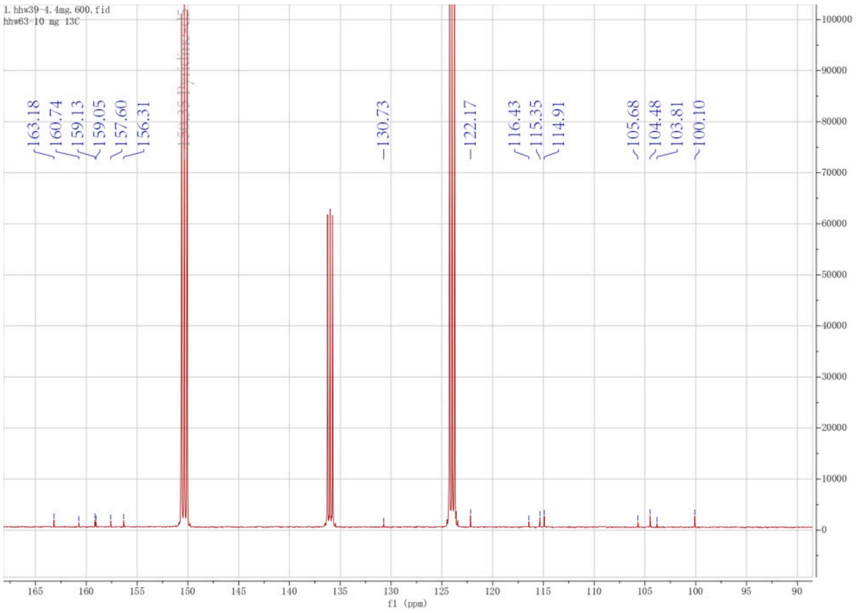

Figure S5. Coumpound 2^{13}C-NMR

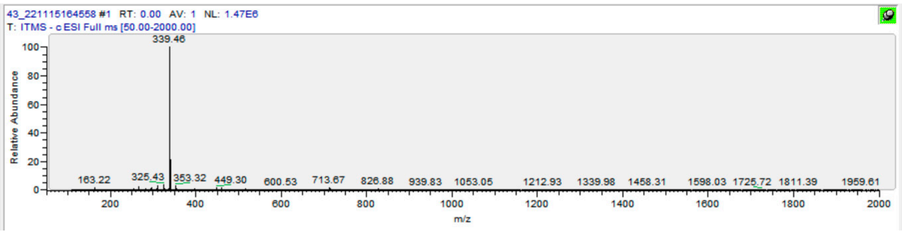

Figure S6. Mass spectra of Coumpound 3

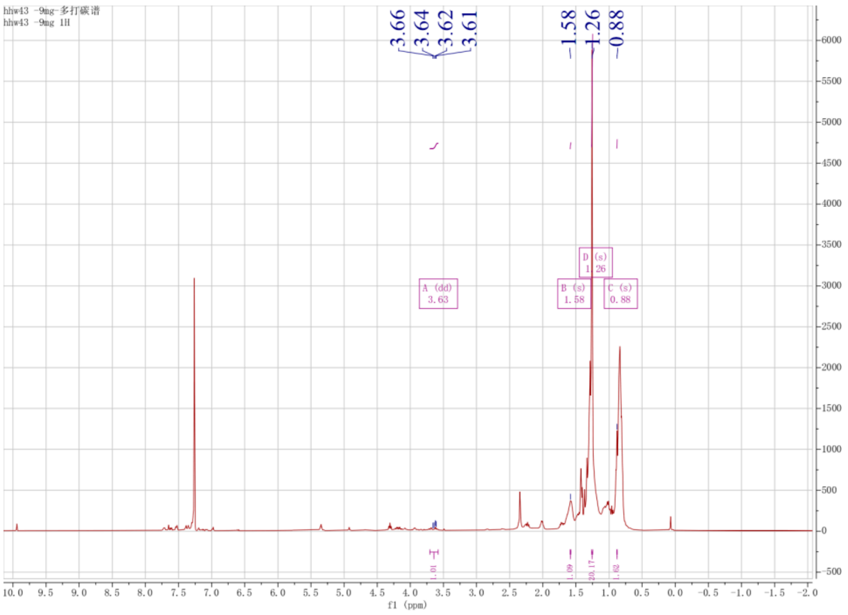

Figure S7. Coumpound 3 ^{1}H-NMR

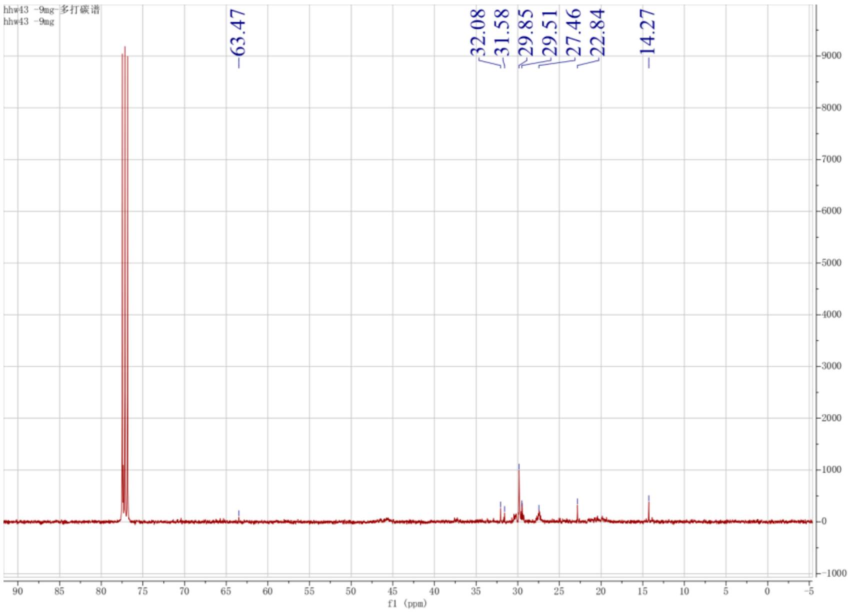

Figure S8. Coumpound 3 ^{13}C-NMR

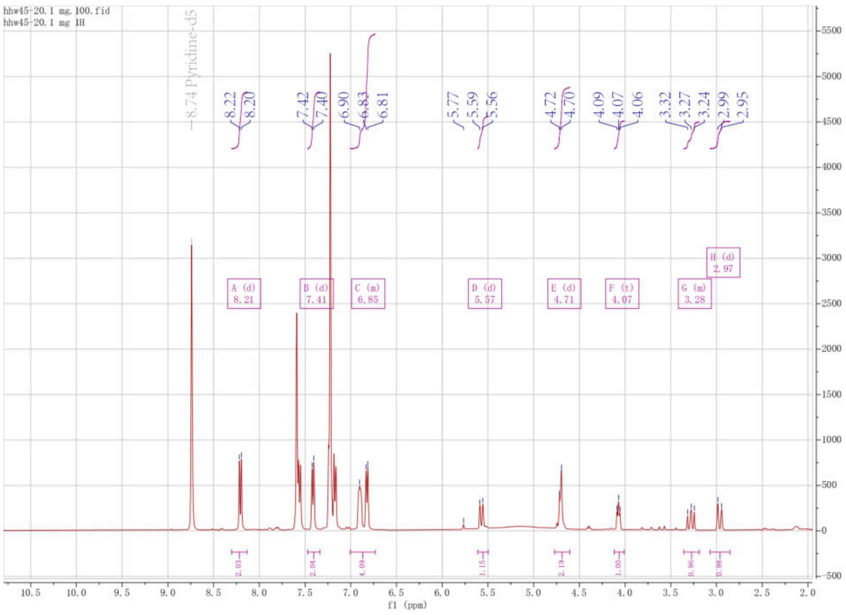

Figure S9. Coumpound 4 ^{1}H-NMR

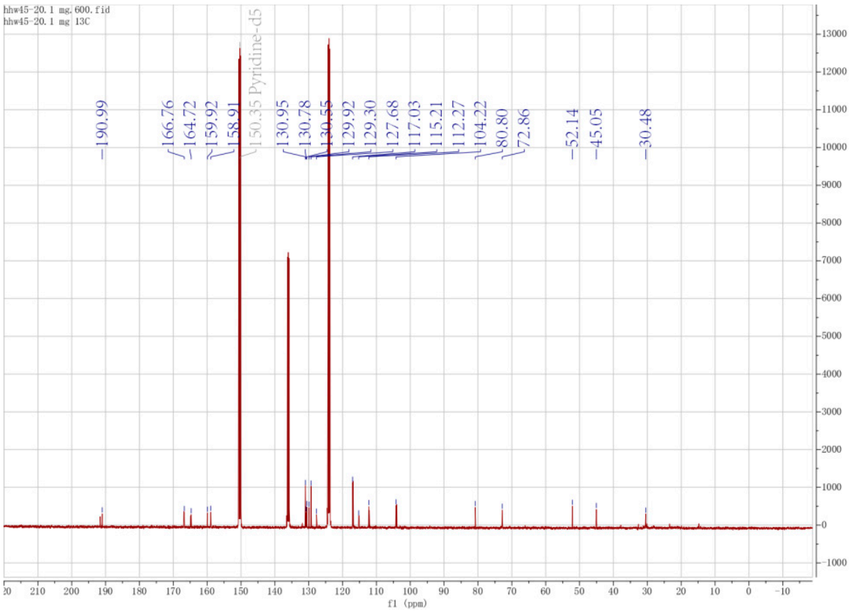

Figure S10. Coumpound 4 ^{13}C-NMR

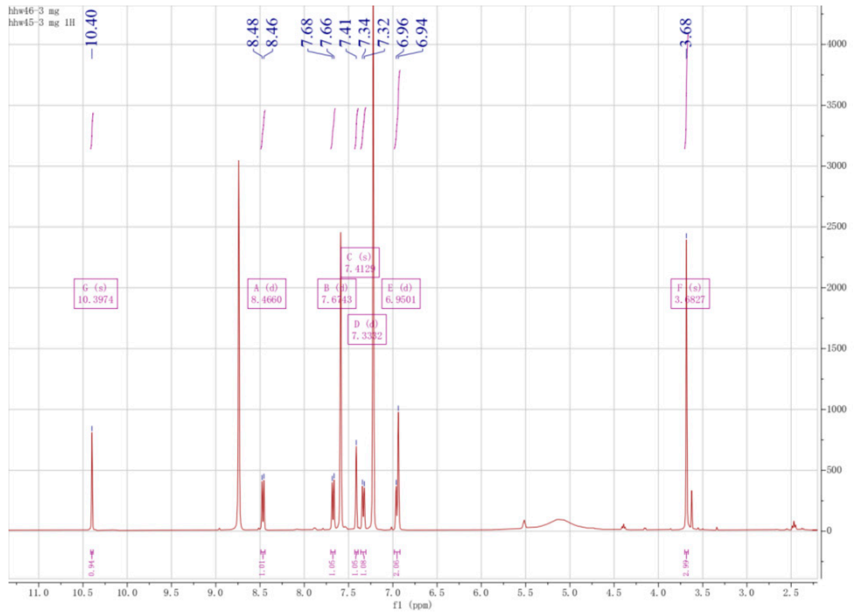

Figure S11. Coumpound 5 ^{1}H-NMR

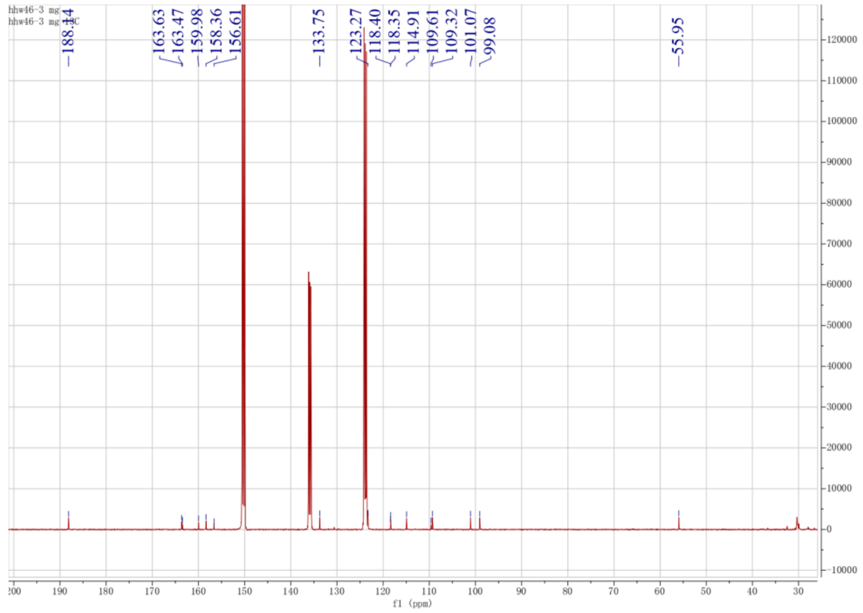

Figure S12. Coumpound 5^{13}C-NMR

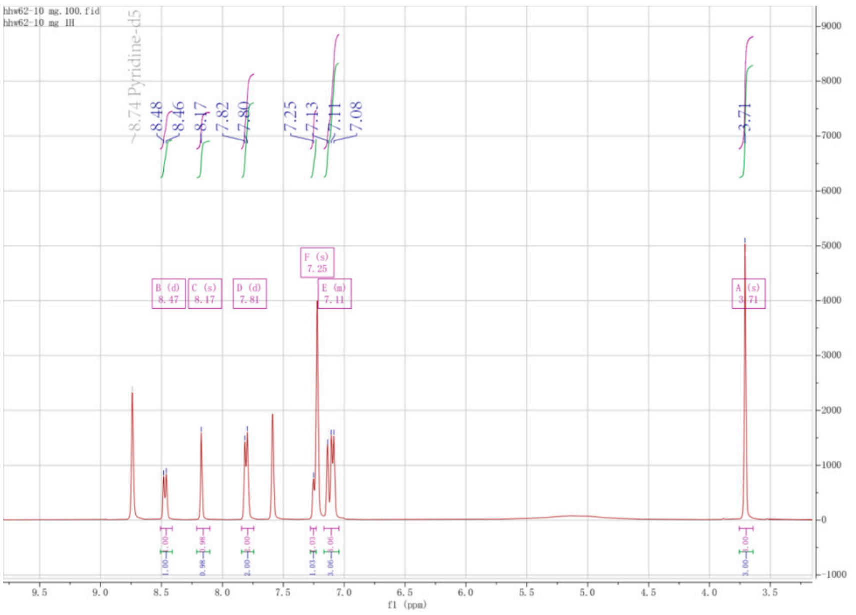

Figure S13. Coumpound 6 ^{1}H-NMR

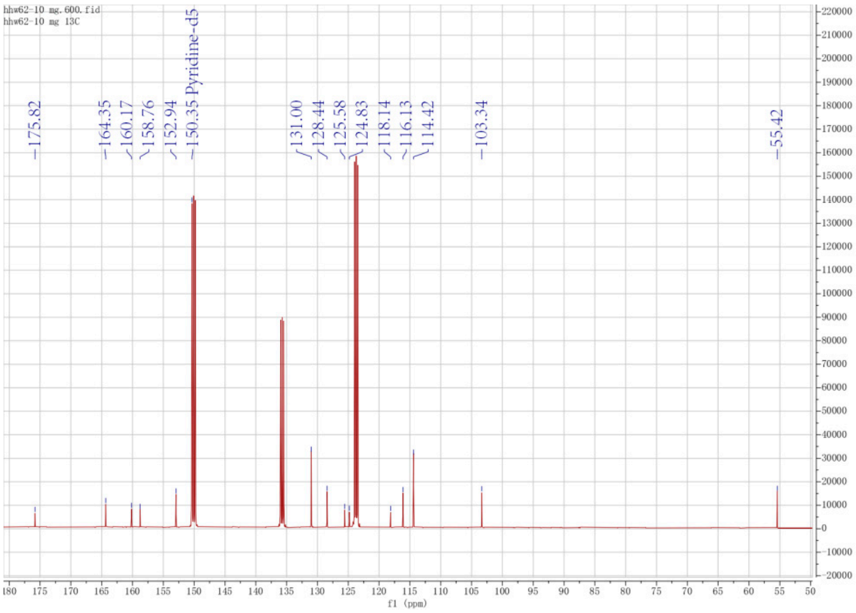

Figure S14. Coumpound 6 ^{13}C-NMR

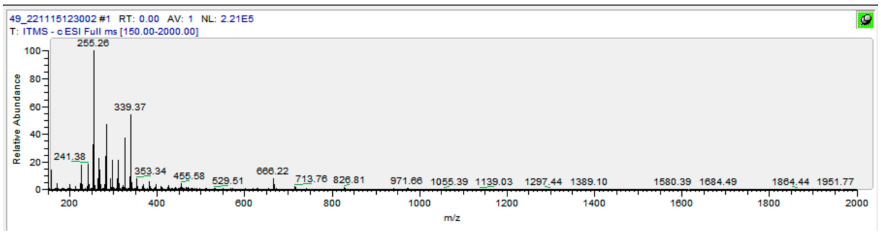

Figure S15. Mass spectra of Coumpound 7

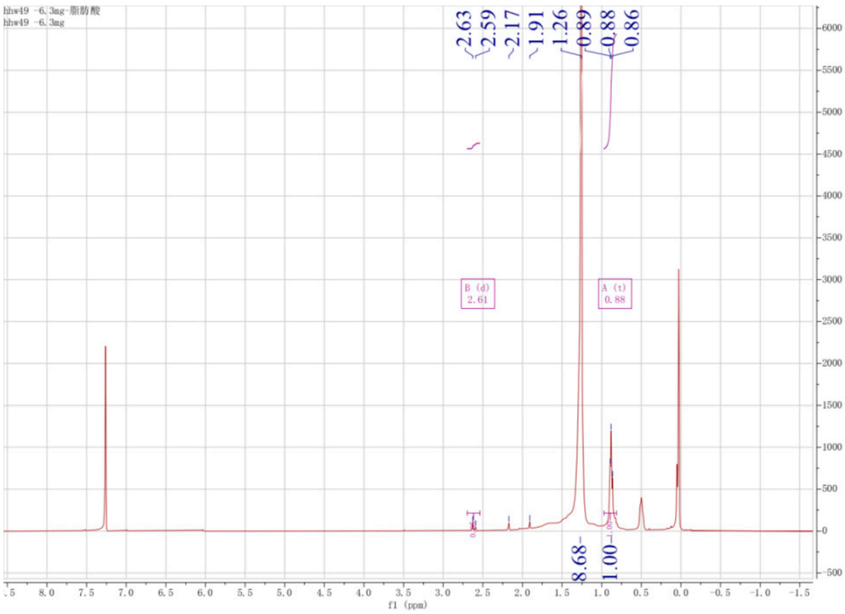

Figure S16. Coumpound 7 ^{1}H-NMR

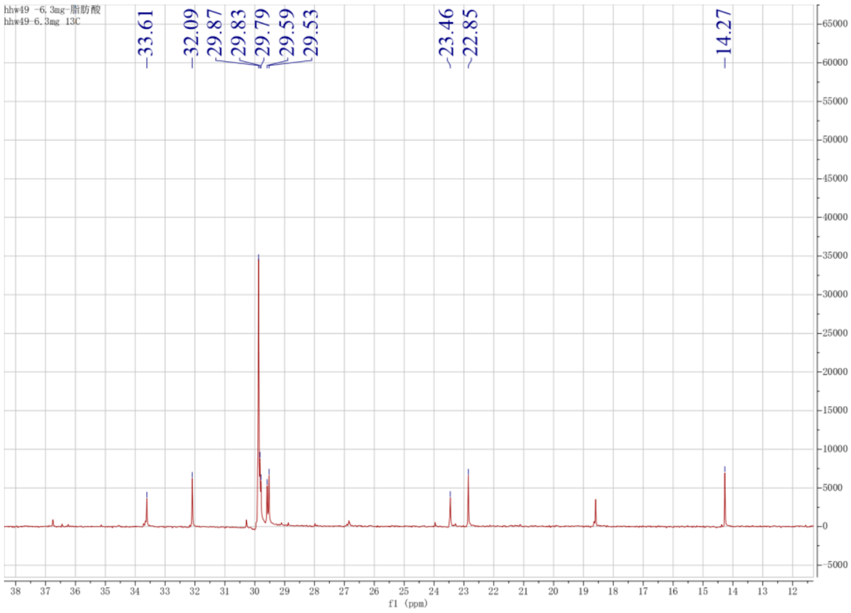

Figure S17. Coumpound 7 ^{13}C-NMR

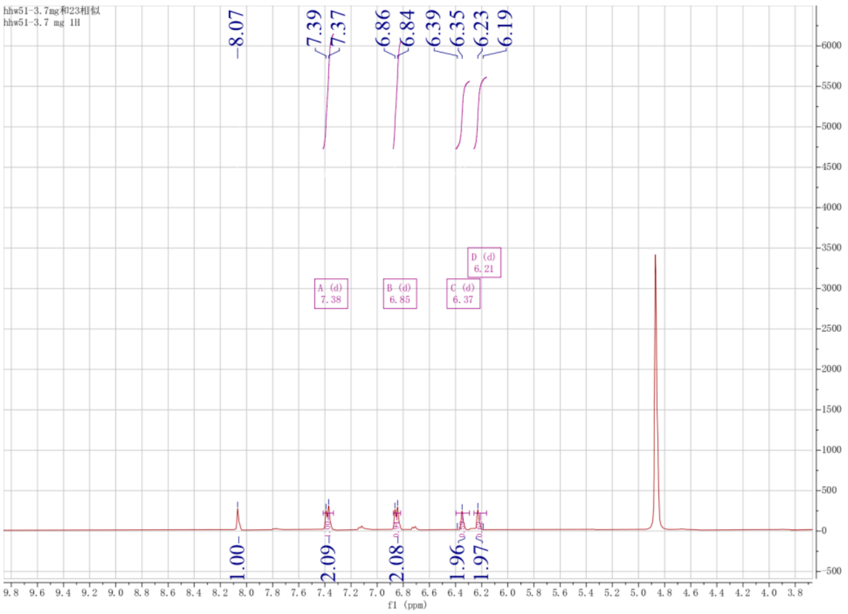

Figure S18. Coumpound 8¹H-NMR

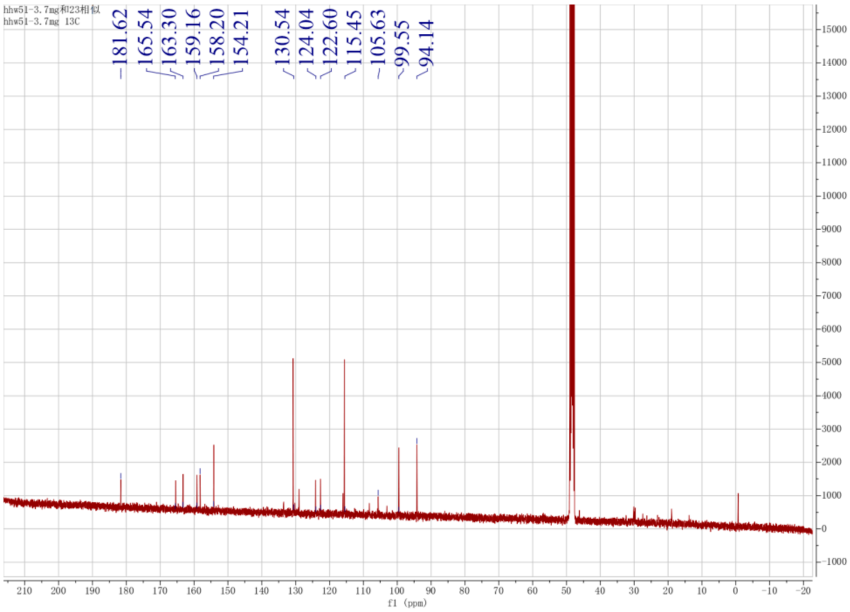

Figure S19. Coumpound 8 ¹³C-NMR

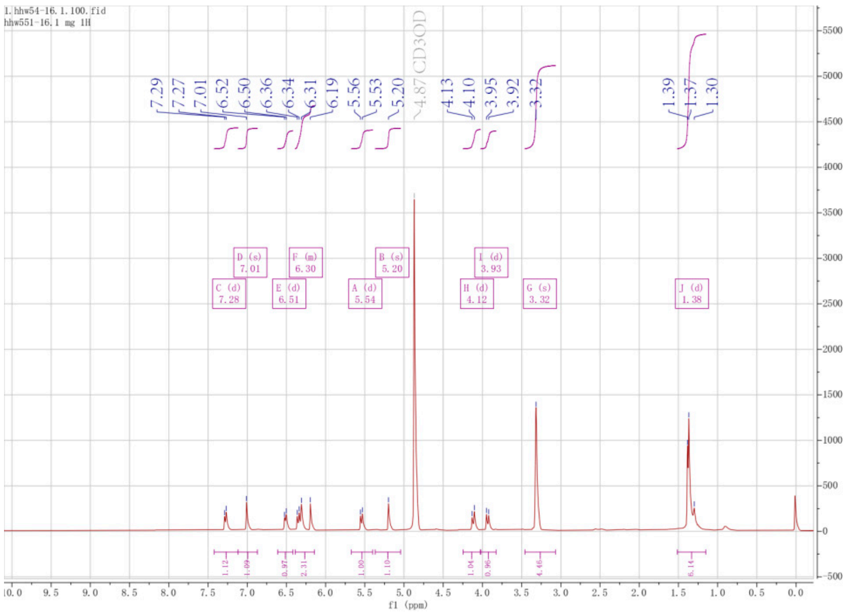

Figure S20. Coumpound 9 ^{1}H-NMR

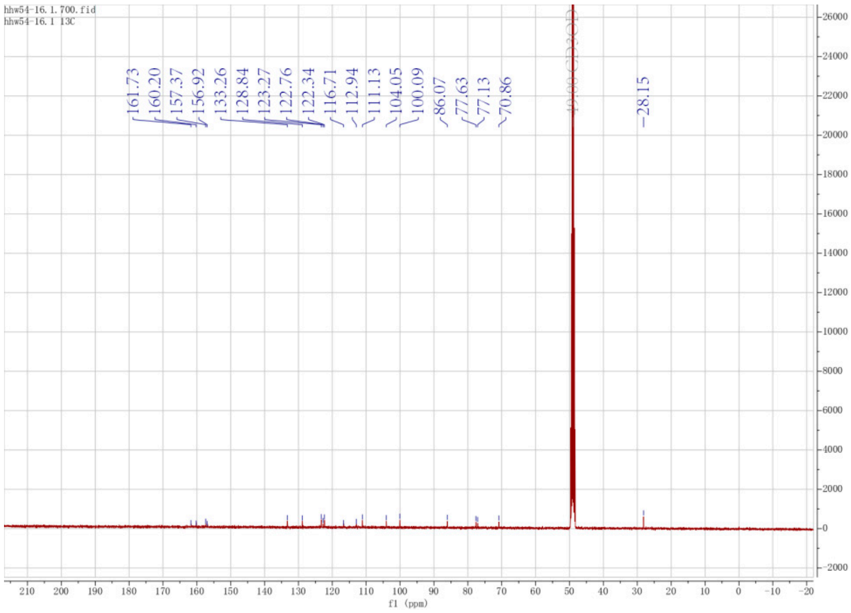

Figure S21. Coumpound 9 ^{13}C-NMR

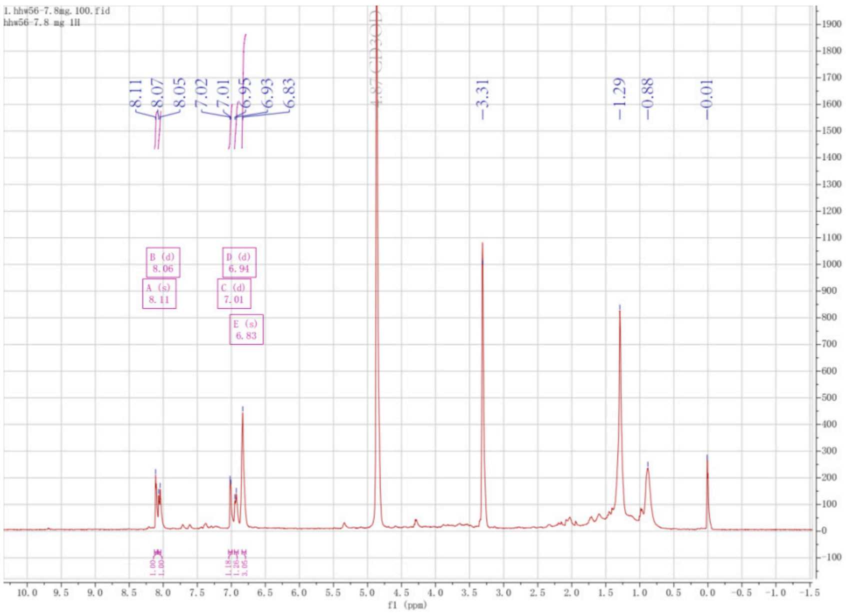

Figure S22. Coumpound 10 ^{1}H-NMR

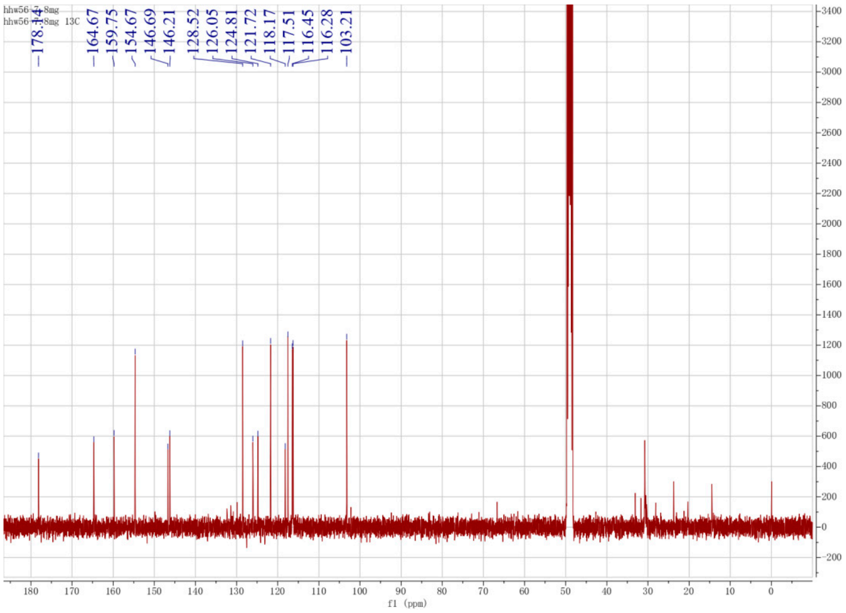

Figure S23. Coumpound 10 ^{13}C-NMR

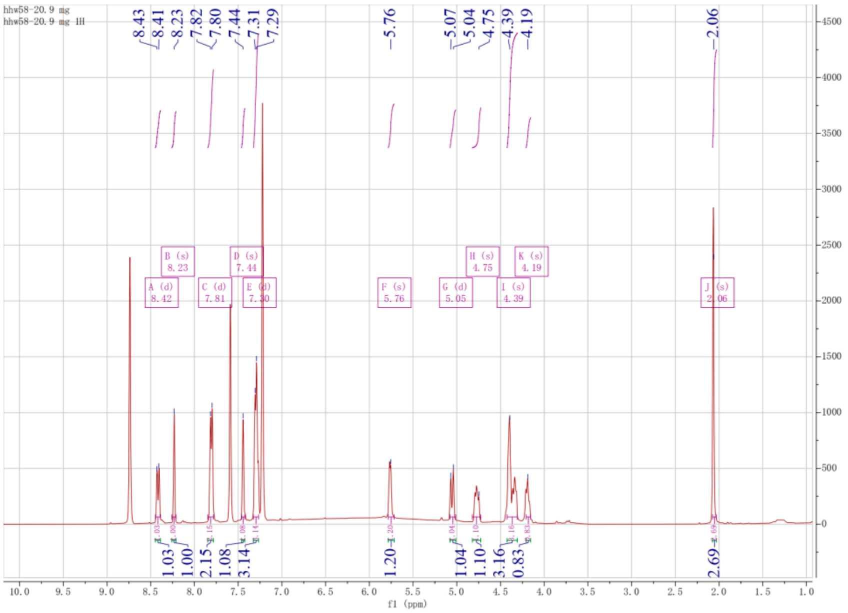

Figure S24. Coumpound 11 ^{1}H-NMR

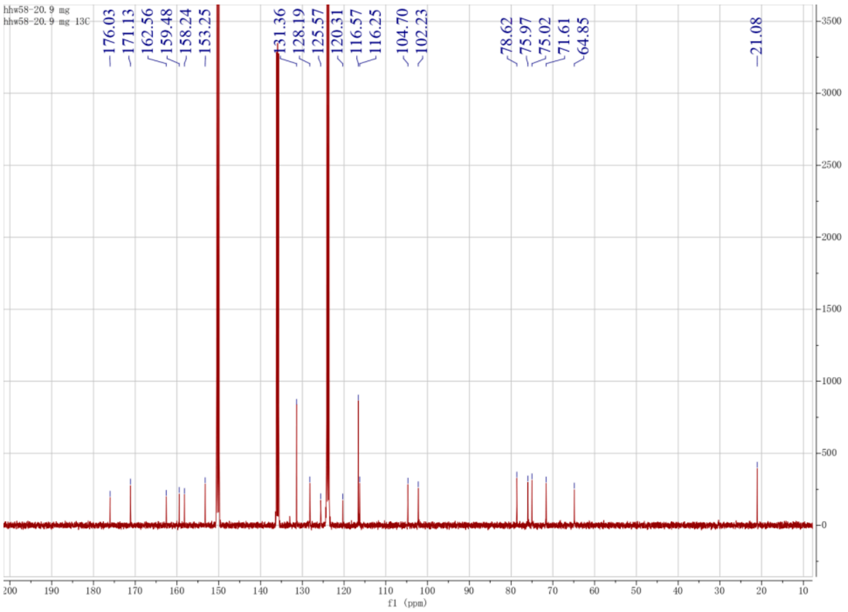

Figure S25. Coumpound 11 ^{13}C-NMR

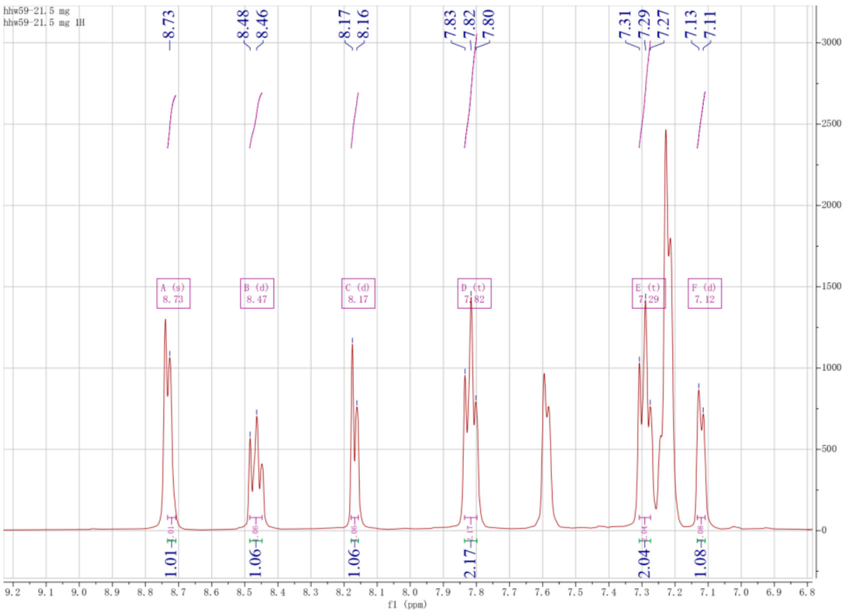

Figure S26. Coumpound 12 ^{1}H-NMR

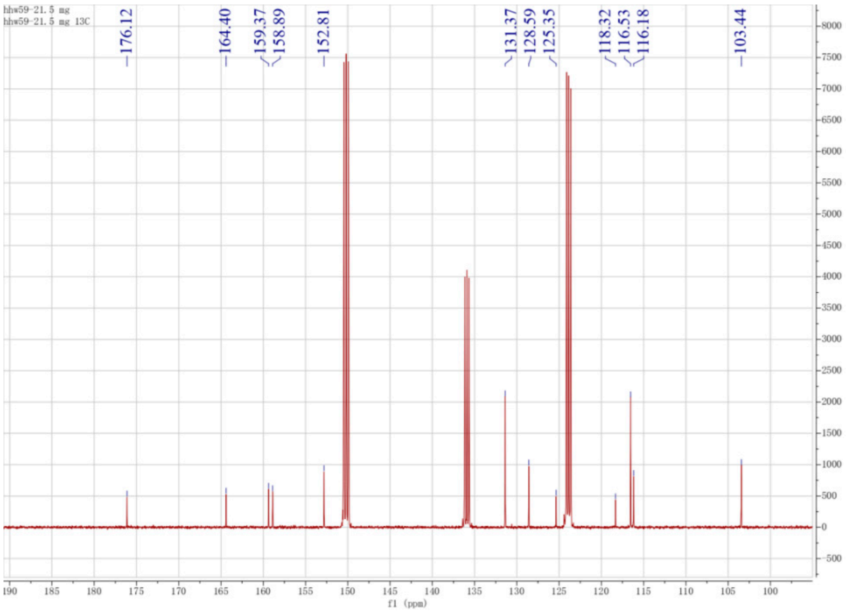

Figure S27. Coumpound 12 ^{13}C-NMR

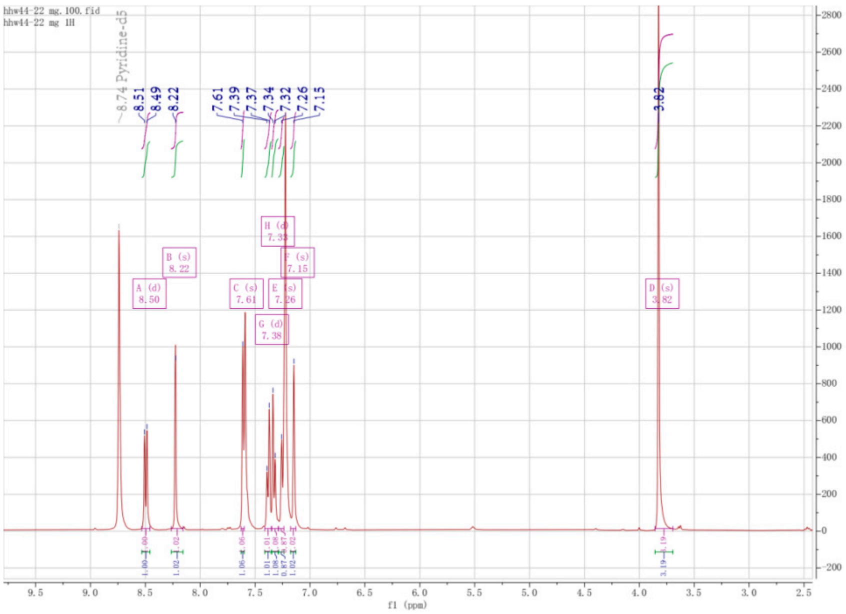

Figure S28. Coumpound 13 ^{1}H-NMR

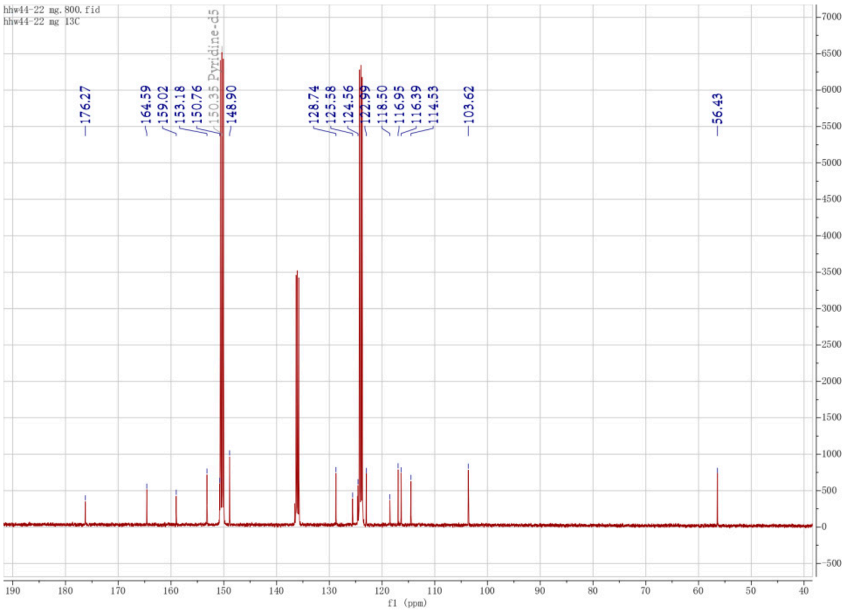

Figure S29. Coumpound 13 ^{13}C-NMR

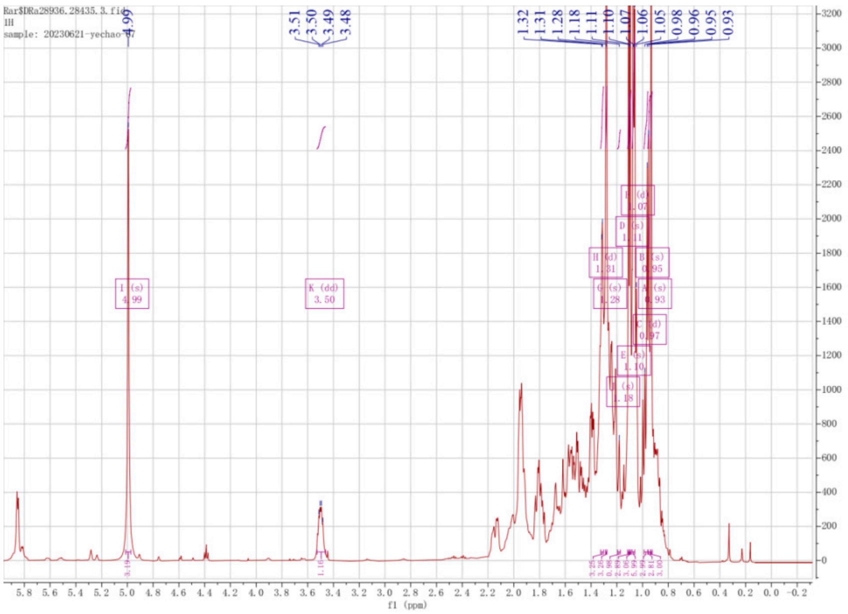

Figure S30. Coumpound 14 ^{1}H-NMR

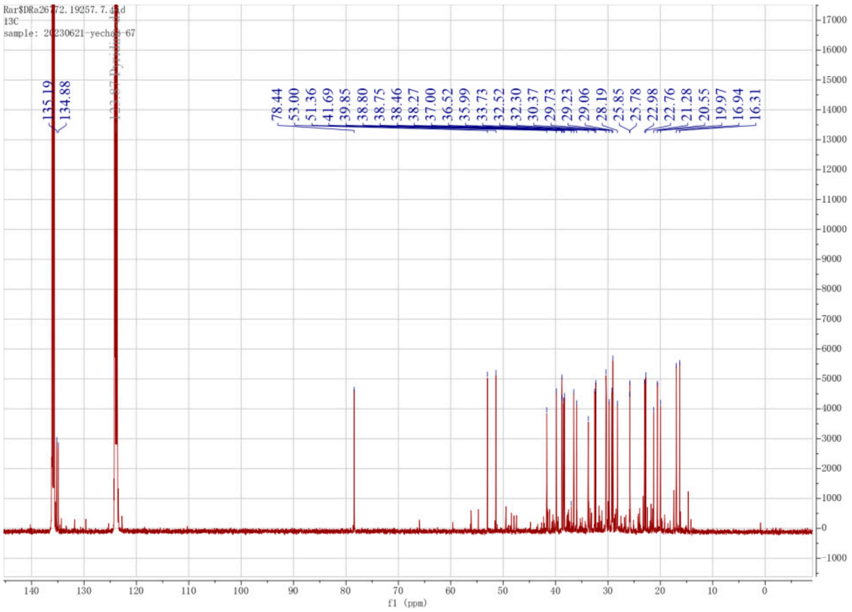

Figure S31. Coumpound 14 ^{13}C-NMR

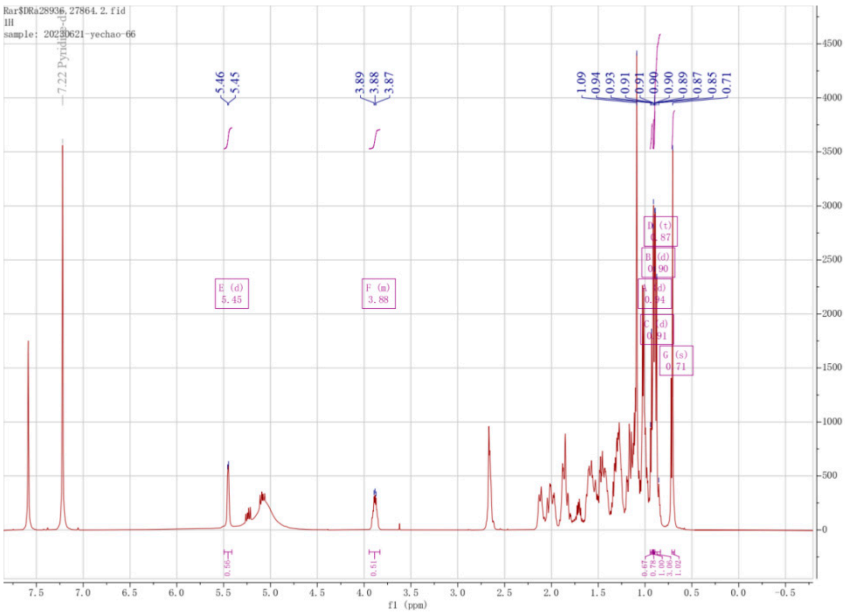

Figure S32. Coumpound 15 ^{1}H-NMR

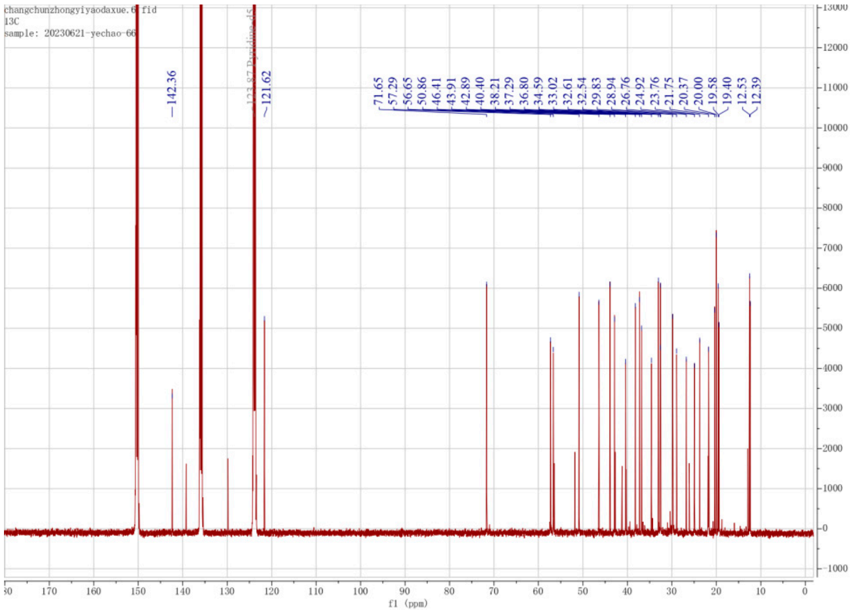

Figure S33. Coumpound 15 ^{13}C-NMR

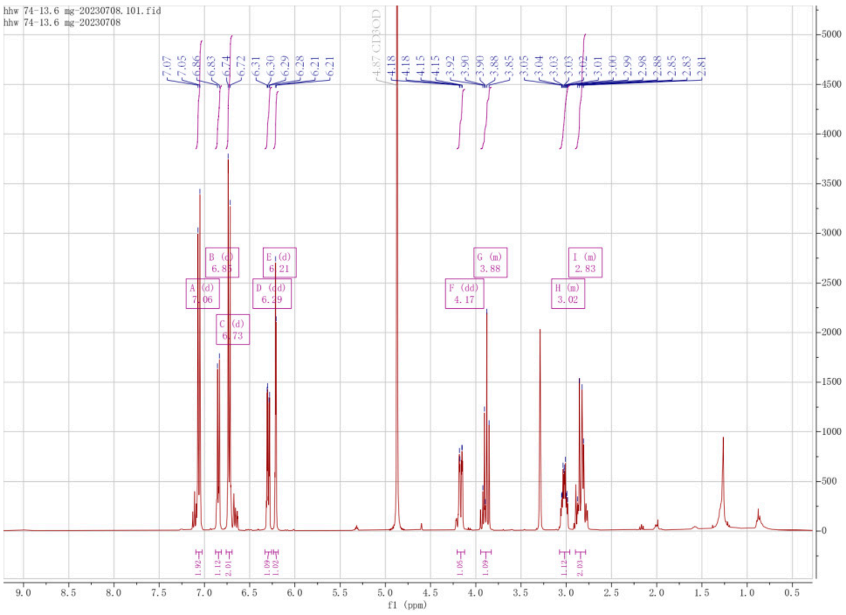

Figure S34. Coumpound 16 ^{1}H-NMR

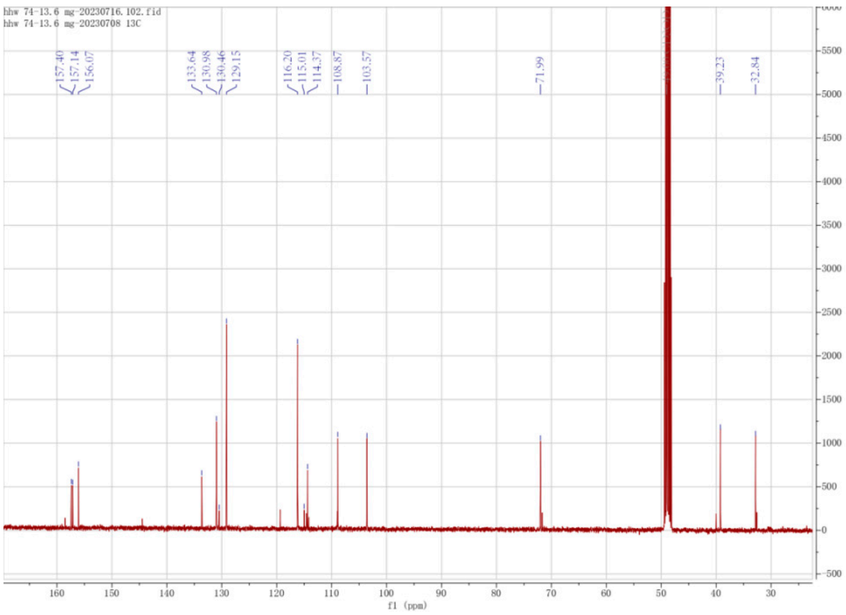

Figure S35. Coumpound 16 ^{13}C-NMR

Compound 1: MS m/z: 339.30 [M-H]-. In 1H-NMR (400 MHz, CDCl3) , δ: 5.35 (2H, t, J=4.7 Hz, H-13), 2.22 (2H, t, J=7.6 Hz, H-2), 2.01 (2H, d, J=5.7 Hz), 1.75-1.53 (4H, m, H-3), 0.88 (3H, t, J=6.4 Hz, H-22). H-22); in the 13C-NMR (100 MHz, CDCl3) , δ: 175.39 (C-1), 130.04 (C-13), 130.07 (C-14), 33.61 (C-2), 23.44 (C-3), 27.37 (C-12, 15), 32.08 (C-20), 29.93-29.0 (C-4~C-11, C-16~C-19), 22.84 (C-21), 14.26 (C-22). Compound 1 was identified as Erucic Acid by comparison with literature [1].

Compound 2: In 1H-NMR (400MHz, C5D5N) , δ: 8.17 (1H, d, J=8.4Hz, H-5), 7.92 (1H, d, J=8.3Hz, H-6'), 7.50 (1H, d, J=8.3Hz, H-3'), 7.36 (1H, d, J=8.3Hz, H-8). In the 13C-NMR (100 MHz, C5D5N), δ: 163.18 (C-2), 160.74 (C-4), 159.13 (C-10), 159.05 (C-4'), 157.60 (C-2'), 156.31 (C-7), 122.17 (C-5), 116.43 (C-5'). 115.35 (C-1'), 114.91 (C-6), 105.6 (C-9), 104.3 (C-3), 103.81 (C-8), 100.10 (C-3'). Compound 2 was identified as 3-(2', 4'-Dihydroxyphenyl)-4, 7-dihydroxy-2H-1-benzopyran-2-one by comparison with literature [2].

Compound 3: MS m/z: 339.46 [M+H]+. In 1H-NMR (400MHz, CDCl3) , δ: 3.63 (2H, dd, J=6.2Hz, J=13.6Hz, H-1), 1.58 (2H, s, H-2), 1.26 (2H×20, S, H-3~H-22), 0.88 (3H, s, H-23). In 13C-NMR (100 MHz, CDCl3), δ: 63.47 (C-1), 32.08 (C-2), 31.58 (C-21), 29.85 (C-4~C-19), 29.51 (C-20), 27.46 (C-3), 22.84 (C-22), 14.27 (C-23). Compound 3 was identified as Tricosanol by comparison with literature [3].

Compound 4: In 1H-NMR (400 MHz, C5D5N) spectrum, δ: 7.67 (1H, d, J=8.1 Hz, H-5), 7.41 (2H, d, J=8.1 Hz, H-2', 6'), 7.17 (2H, d, J=8.0 Hz, H-3', 5'), 6.90 (1H, dd, J=7.1, 3.0 Hz , H-6), 6.82 (1H, d, J=7.6 Hz, H-8), 5.57 (1H, m, H-2), 3.26 (1H, dd, J=26.1, 12.8 Hz, H-3β), 2.97 (1H, m, H-3α). In the 13C-NMR (100 MHz, C5D5N) spectrum, δ: 191.57 (C=O), 166.85 (C-7), 164.84 (C-9), 159.9 (C-4'), 130.94 (C-1'), 129.92 (C-5), 129.30 (C-2',6'), 116.87 (C-3',5'), 115.21 (C-10), 112.27 (C-6), 103.98 (C-8), 80.80 (C-2), 45.00 (C-3). Compound 4 was identified as (2S)-4'-hydroxy-7'-methoxyflavanone by comparison with literature [4].

Compound 5: In the 1H-NMR (400 MHz, C5D5N) spectrum, δ: 10.40 (1H, s, 3-CHO), 8.47 (1H, d, J=8.4Hz, H-4), 7.67 (1H, d, J=8.1Hz, H-6), 7.41 (1H, s, H-7), 7.33 (1H, d, J=8.4Hz, H-5). 6.96 (1H, s, H-3), 6.94 (1H, s, H-5), 3.68 (3H, s, 2-OCH3); in the 13C-NMR (100 MHz, C5D5N) spectrum, δ: 188.14 (CHO), 163.63 (C-2), 163.47 (C-11), 159.98 (C-9), 158.36 (C-7), 156.61 (C-13), 133.75 (C-3), 123.27 (C-5), 118.4 (C-4), 118.35 (C-15), 114.91 (C-6), 109.61 (C-14), 109.32 (C-10), 101.07 (C-12), 99.08 (C-8), 55.95 (OCH3). Compound 5 was identified as Puerariafuran by comparison with literature [5].

Compound 6: In 1H-NMR (400 MHz, C5D5N) spectrum, δ: 8.47 (1H, d, J=8.6 Hz, H-5), 8.17 (1H, s, H-25), 7.80 (2H, d, J=8.0 Hz, H-2', 6'), 7.11 (4H, m, H-3', 5', 6, 8), 3.71 (3H, s. 4'-OCH3). In the 13C-NMR (100 MHz, C5D5N) spectrum, δ: 175.93 (C-4), 164.53 (C-7), 160.31 (C-4'), 158.89 (C-9), 153.13 (C-2), 131.16 (C-2', 6'), 128.59 (C-5), 125.73 (C-1'), and 124.97 (C-3), 118.28 (C-10), 116.30 (C-6), 114.57 (C-3', 5'), 103.50 (C-8), 55.57 (4'-OCH3). Compound 6 was identified as Formononetin by comparison with literature [6].

Compound 7: MS m/z: 255.26 [M+H]+. In the 1H-NMR (400 MHz, CDCl3) spectrum, δ: 2.61 (1H, d, J = 14.5 Hz, H-2), 0.88 (1H, t, J = 6.1 Hz, H-1). In the 13C-NMR (100 MHz, CDCl3) spectrum, δ: 33.61 (C-1), 23.46 (C-2), 29.53-29.87 (C-3~C-14), 32.09 (C-15), 22.85 (C-16), 14.27 (C-17). Compound 7 was identified as Heptaecanoic Acid by comparison with literature [7].

Compound 8: In 1H-NMR (400 MHz, CD3OD) spectrum, δ: 8.07 (1H, s, H-2), 7.38 (2H, d, J=8.3 Hz, H-2', H-6'), 6.85 (2H, d, J=8.2 Hz, H-3', 5'), 6.37 (1H, d, J=14.8 Hz, H-8), 6.21 (1H,

d, J=16.2 Hz, H-6). In the 13C-NMR (100 MHz, CD3OD) spectrum, δ: 181.62 (C-4), 165.54 (C-7), 163.30 (C-5), 159.16 (C-4'), 158.20 (C-9), 154.21 (C-2), 130.54 (C-2', C-6'), 124.04 (C-1'), 122.60 (C-3), 115.45 (C-3', C-5'), 105.63 (C-10), 99.55 (C-6), 94.14 (C-8). Compound 8 was identified as Genistein by comparison with literature [8].

Compound 9: In the 1H-NMR (400 MHz, CD3OD) spectrum, δ: 7.28 (d, J=8.4 Hz, 1H, H-5), 7.01 (s, 1H, H-6'), 6.51 (1H, d, J=9.3 Hz, H-6), 6.35 (1H, d, J=9.5 Hz, H-4''), 6.31 (d, 1H, H 8), 6.19 (s, 1H, H-3''), 5.54 (1H, d, J=10.1 Hz, H-3''), 5.20 (s, 1H, H-4), 4.12 (d, J=11.3 Hz, 1H, H-2''a), 3.93 (d, J=11.6 Hz, 1H, H2''b), 1.39 (s, 3H, 2''a-C- H3), 1.37 (s, 3H, 2''b-C-H3). In the 13C-NMR (100 MHz, CD3OD) spectrum, δ: 161.73 (C-1'), 160.20 (C-7), 157.37 (C-9), 156.92 (C-5'), 133.26 (C-5), 128.84 (C-3''), 123.27 (C-2'), 122.76 (C-3'), and 122.34 (C-4''), 116.71 (C-4'), 112.94 (C-10), 111.13 (C-6), 104.05 (C-8), 100.09 (C-6'), 86.07 (C-4), 77.63 (C-3), 77.13 (C-2''), 70.86 (C-2), 28.15 (2' 'a-CH3, 2''b-CH3). Compound 9 was identified as Tuberosin by comparison with literature [9].

Compound 10: In 1H-NMR (400 MHz, CD3OD) spectrum, δ: 8.11 (1H, d, J=4.1Hz, H-2), 8.05 (1H, d, J=8.8, H-5,), 7.01 (1H, d, J=3.6Hz, H-2'), 6.94 (1H, d, J=7.9Hz, H-6), 6.83 (3H, s, H-8, 5', 6'). In the 13C-NMR (100 MHz, CD3OD) spectrum, δ: 103.21 (C-8), 116.28 (C-6), 116.45 (C-5'), 117.51 (C-2'), 118.17 (C-10), 121.72 (C-6'), 124.81 (C-1'), 126.05 (C-3), and 128.52 (C-5), 146.21 (C-3'), 146.69 (C-4'), 154.67 (C-2), 159.75 (C-9), 164.67 (C-7), 178.14 (C-4). Compound 10 was identified as 3',4',7-Trihydroxy Isoflavone by comparison with literature [10].

Compound 11: In 1H-NMR (400MHz, C5D5N) spectrum, δ: 8.42 (IH, d, J=8.8Hz, H-5), 8.23 (1H, s, H-2), 7.81 (2H, d, J=7.1Hz, H-2', 6'), 7.44 (1H, s, H-8), 7.30 (1H, d, J=6.3Hz. H-6, H-3', 5'), 5.76 (1H, s, The upper end of the sugar matrix), 5.07-4.19 (6H, Hydroproton on sugar), 2.06 (3H, s, -OAc). In the 13C-NMR (100 MHz, C5D5N) spectrum, δ: 176.03 (C-4), 171.13 (-C=O), 162.56 (C-7), 159.48 (C-9), 158, 24 (c-4'), 153, 25 (C-2), 131, 36 (C-2′, 6′), 128, 19 (C-5). 125.57 (c-1'), 120.31 (c-10), 116.57 (c-3', 5'), 116.25 (c-6), 104.7 (c-8), 102.23 (c-1''), 78.62 (c-3''), 75.97 (c-5''), 75.02 (c-2''), 71.61 (c-4'') , 64.85 (C-6''), 21.08 (-CH3). Compound 11 was identified as 6''-O-Acetyldaidzin by comparison with literature [11].

Compound 12: In the 1H-NMR (400MHz, C5D5N) spectrum, δ: 8.73 (1H, s, H-2), 8.47 (1H, d, J=7.9Hz, H-5), 8.17 (1H, d, J=5.4Hz, H-6), 7.82 (2H, t, J=6.6Hz, H-2', 6'), 7.29 (2H, t. J=6.6 Hz, H-3', 5'), 7.12 (1H, d, J=5.5 Hz, H-8); in the 13C-NMR (100 MHz, C5D5N) spectrum, δ: 176.12 (C-4), 164.4 (C-7), 159.37 (C-9), 158.89 (C-4'), 152.81 (C-2), and 131.37 (C-2', 6'), 128.59 (C-5), 125.35 (C-1'), 118.32 (C-10), 116.53 (C-3', 5'), 116.18 (C-6), 103.44 (C-8). Compound 12 was identified as Daidzein by comparison with literature [12].

Compound 13: In 1H-NMR (400 MHz, C5D5N) spectrum, δ: 8.50 (1H, d, J=8.8 Hz, H-5), 8.22 (1H, s, H-2), 7.61 (1H, s, H-2'), 7.38 (1H, d, J=8.3 Hz, H-6), 7.33 (1H, d, J=8.1 Hz, H-5 '), 7.26 (1H, s, H-6'), 7.15 (1H, s, H-8), 3.82 (3H, s, OCH3). In the 13C-NMR (100 MHz, C5D5N) spectrum, δ: 176.27 (C-4), 164.59 (C-7), 159.02 (C-9), 153.18 (C-2), 150.76 (C-4'), 148.90 (C-3'), 128.74 (C-5), 125.58 (C-1'), and 124.56 (C-3), 122.99 (C-6'), 118.50 (C-5'), 116.95 (C-10), 116.39 (C-6), 114.53 (C-2'), 103.62 (C-8), 56.44 (O-CH3). Compound 13 was identified as 7,4'-Dihydroxy-3'-methoxyisoflavone by comparison with literature [13].

Compound 14: In 1H-NMR (400 MHz, C5D5N) spectrum, δ: 0.93 (3H, s, H-24), 0.95 (3H, s, H-27), 0.97 (3H, d, j=9.0 Hz, H-30), 1.07 (6H, d, J=5.2 Hz, H-23, H-26), 1.10 (3H, s, H- 25), 1.11 (3H, s, H-29), 1.28 (3H, s, H-28), 3.50 (1H, dd, j=5.3, 10.7 Hz, H-3). In the 13C-NMR (100

MHz, C5D5N) spectrum, δ: 135.19 (C-8), 134.88 (C-9), 78.44 (C-3), 53.00 (C-18), 51.36 (C-5), 41.69 (C-14), 39.85 (C-28), 38.80 (C-13), 38.75 (C-16), 38.46 (C-4), 38.27 (C-10), 36.52 (C-20), 35.99 (C-1), 33.73 (C-22), 32.30 (C-17), 30.37 (C-19), 30.28 (C-21), 29.23 (C-12), 29.06 (C-23), 28.19 (C-7), 28.22 (C-29), 25.85 (C-15), 25.78 (C-2), 22.98 (C-30), 22.76 (C-26), 21.28 (C-11), 20.55 (C-25), 19.97 (C-6), 16.94 (C-24), 16.31 (C-27). Compound 14 was identified as Isobartriol by comparison with literature [14].

Compound 15: In the 1H-NMR (400MHz, C5D5N) spectrum, δ: 5.45 (1H, d, J=5.0Hz, H-6), 3.88 (1H, m, H-3), 0.94 (3H, s, H-19), 0.90 (3H, d, J=5.9Hz, H-26), 0.94 (3H, d, J=2.4Hz, H-27), 0.90 (3H, d, J=1.8 Hz, H-21), 0.87 (3H, t, J=9.5 Hz, H-29), 0.71 (3H, s, H-18). In the 13C-NMR (100 MHz, C5D5N) spectrum, δ: 142.36 (C-5), 121.62 (C-6), 71.65 (C-3), 57.29 (C-14), 56.65 (C-17), 50.86 (C-9), 46.41 (C-24), 43.91 (C-4), 42.89 (C-13), 40.40 (C-12), 38.21 (C-1), 37.29 (C-10), 36.80 (C-20), 34.59 (C-22), 33.02 (C-7), 32.61 (C-8), 32.54 (C-2), 29.83 (C-23), 28.94 (C-16), 26.76 (C-25). 24.92 (C-15), 23.76 (C-28), 21.75 (C-11), 20.37 (C-27), 20.00 (C-19), 19.58 (C-21), 19.40 (C-26), 12.53 (C-18), 12.39 (C-29). Compound 15 was identified as β-sitosterol by comparison with literature [15].

Compound 16: In 1H-NMR (400 MHz, CD3OD) spectrum, δ: 7.06 (2H, d, J=8.6 Hz, H-2', 6'), 6.85 (1H, d, J=8.2 Hz, H-6), 6.73 (d, J=8.5 Hz, 2H, H-2', 6'), 6.29 (dd, J=8.2, 2.6 Hz. 1H, H-5), 6.21 (d, J=2.5 Hz, 1H, H-8), 4.17 (dd, J=10.5, 1.7 Hz, 1H, H-2), 3.94-3.83 (m, 1H, H-3), 3.07-2.96 (m, 1H, H-4), 2.90-2.79 (m, 2H, H-9). In the 13C-NMR (100 MHz, CD3OD) spectrum, δ: 71.99 (C-2), 39.23 (C-3), 32.84 (C-4), 133.64 (C-5), 108.87 (C-6), 157.4 (C-7), 103.57 (C-8), 157.14 (C-9), 114.37 (C 10), 130.98 (C-1'), 130.46 (C-2'), 116.2 (C-3'), 156.07 (C-4'), 115.01 (C-5'), 129.15 (C-6'). Compound 16 was identified as 7,4'-Dihydroxyhomoisoflavane by comparison with literature [16].

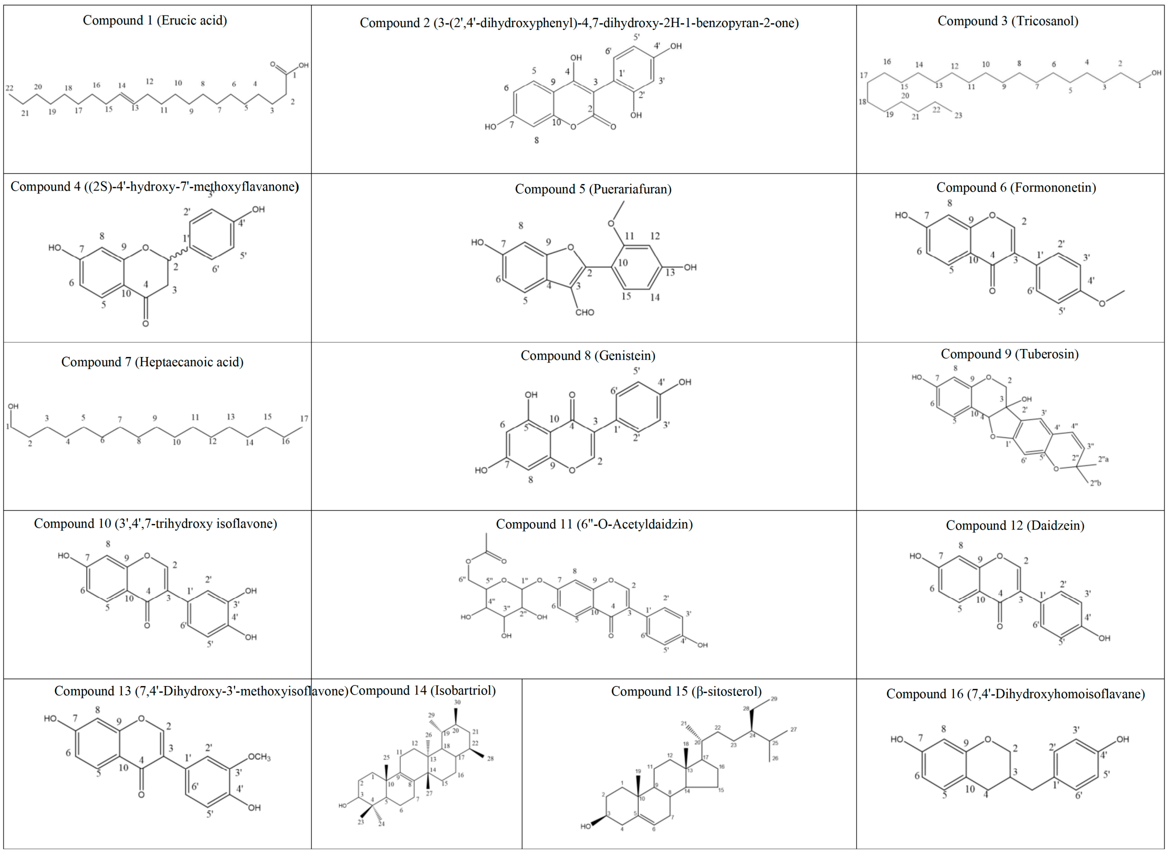

Figure S36. Structure of compounds 1-16

Table S1 Anti-inflammatory targets of formononetin

Number	Target name	Degree	Number	Target name	Degree
1	TNF	40	37	ALOX15	8
2	EGFR	33	38	BCL2	8
3	PTGS2	32	39	BIRC5	8
4	HSP90AA1	30	40	PLG	8
5	ESR1	28	41	TYK2	7
6	PPARG	26	42	TLR9	7
7	MAPK14	18	43	NOX4	7
8	PPARA	18	44	PON1	6
9	MCL1	17	45	ABCG2	6
10	HSP90AB1	16	46	TYR	6
11	ABCB1	15	47	ADORA1	6
12	PTPN1	14	48	PTGER3	6
13	GSK3B	14	49	RXRA	6
14	PRKACA	14	50	OPRD1	5
15	IL2	14	51	ALDH2	5
16	ABL1	13	52	ALOX12	5
17	CDK2	13	53	BAD	5
18	PLA2G4A	12	53	PIM1	5
19	PTGS1	12	55	MIF	5
20	LRRK2	12	56	ESRRB	5
21	RAF1	12	57	MAOA	4
22	NOS2	12	58	ADORA2A	4
23	ALOX5	11	59	PTGER1	4
24	ESR2	11	60	XDH	4
25	AKR1B1	10	61	PTGDR	4
26	KIT	10	62	PTGER2	4
27	ADRB2	10	63	STS	3
28	SLC6A4	9	64	EPHB4	2
29	ACHE	9	65	IKBKE	2
30	BACE1	9	66	PFKFB3	1
31	CYP19A1	9	67	PRSS1	1
32	EZR	8	68	CAR	0
33	CYP1B1	8	69	ERCC5	0
34	CCNA2	8			
35	MAOB	8			
36	DPP4	8			

Table S2 Intersecting target protein interactions

Node1	Node2	Co-expression	Experimentally Determined interaction	Database annotated	Automated Text mining	Combined score
CDK2	CCNA2	0.401	0.999	0.9	0.995	0.999
EGFR	PTPN1	0	0.893	0.9	0.988	0.999
EGFR	HSP90AA1	0	0.884	0.9	0.989	0.999
LRRK2	HSP90AA1	0	0.919	0	0.989	0.999
HSP90AA1	ESR1	0	0.887	0.6	0.989	0.999
PPARG	RXRA	0.062	0.981	0.9	0.99	0.998
EGFR	ESR1	0	0.696	0	0.988	0.996
BAD	BCL2	0	0.905	0.9	0.559	0.995
HSP90AA1	HSP90AB1	0.781	0.888	0.8	0.883	0.994
HSP90AA1	PPARA	0	0.51	0.9	0.869	0.993
PPARA	RXRA	0.062	0.885	0.9	0.989	0.991
RAF1	BAD	0	0.876	0.9	0.259	0.99
ESR2	ESR1	0	0.873	0.9	0.991	0.987
PTGS2	ALOX5	0.081	0	0.9	0.828	0.982
PPARG	TNF	0	0	0.9	0.807	0.979
NOS2	TNF	0.063	0	0.9	0.796	0.979
PRKACA	BAD	0	0.729	0.9	0.232	0.977
MAPK14	TNF	0	0	0.9	0.768	0.975
HSP90AA1	ABL1	0	0.829	0	0.856	0.974
PTGS1	ALOX5	0.096	0	0.9	0.728	0.973
PTGS1	ALOX15	0.062	0	0.9	0.61	0.96
PTPN1	TYK2	0.062	0.298	0.9	0.452	0.959
PLG	DPP4	0.062	0	0	0.957	0.958
MCL1	BCL2	0	0.492	0.9	0.605	0.958
PTPN1	ABL1	0.062	0.426	0.9	0.308	0.957
MAPK14	ESR1	0	0.264	0.9	0.458	0.956
HSP90AA1	MCL1	0	0.128	0.9	0.536	0.956
ADRB2	EZR	0	0	0.9	0.553	0.953
PRKACA	GSK3B	0.079	0.721	0.8	0.276	0.953
TNF	IKBKE	0.115	0.866	0	0.637	0.953
ALOX12	PTGS2	0.062	0	0.9	0.527	0.951
NOX4	PTPN1	0.049	0	0.9	0.528	0.951
PRKACA	HSP90AA1	0	0.654	0.9	0.188	0.969
PPARA	TNF	0	0	0.9	0.7	0.968
CCNA2	BIRC5	0.921	0.144	0	0.559	0.967

EGFR	HSP90AB1	0	0.89	0	0.699	0.965
HSP90AA1	ESR2	0	0.285	0.6	0.886	0.964
NOS2	HSP90AA1	0.062	0.299	0.9	0.513	0.963
ALOX12	PTGS1	0.107	0	0.9	0.603	0.961
ALDH2	MAOA	0.064	0	0.9	0.623	0.961
PTGS2	ALOX15	0.062	0	0.9	0.622	0.961

Table S3 GO annotation enrichment analysis

GO	Category	Description	Count
GO:0006954	GO Biological Processes	inflammatory response	15
GO:0033138	GO Biological Processes	positive regulation of peptidyl-serine phosphorylation	9
GO:0009410	GO Biological Processes	response to xenobiotic stimulus	10
GO:0001666	GO Biological Processes	response to hypoxia	9
GO:0008630	GO Biological Processes	intrinsic apoptotic signaling pathway in response to DNA damage	6
GO:0043066	GO Biological Processes	negative regulation of apoptotic process	12
GO:0043065	GO Biological Processes	positive regulation of apoptotic process	10
GO:0046777	GO Biological Processes	protein autophosphorylation	8
GO:0019369	GO Biological Processes	arachidonic acid metabolic process	5
GO:0097192	GO Biological Processes	extrinsic apoptotic signaling pathway in absence of ligand	5
GO:0005886	GO Cellular Components	plasma membrane	37
GO:0005739	GO Cellular Components	mitochondrion	18
GO:0048471	GO Cellular Components	perinuclear region of cytoplasm	13
GO:0005829	GO Cellular Components	cytosol	36
GO:0032991	GO Cellular Components	macromolecular complex	12
GO:0045121	GO Cellular Components	membrane raft	8
GO:0016324	GO Cellular Components	apical plasma membrane	9
GO:0005741	GO Cellular Components	mitochondrial outer membrane	7
GO:0005654	GO Cellular Components	nucleoplasm	28
GO:0005768	GO Cellular Components	endosome	8
GO:0042803	GO Molecular Functions	protein homodimerization activity	18
GO:0019899	GO Molecular Functions	enzyme binding	13
GO:0016702	GO Molecular Functions	oxidoreductase activity, acting on	5

		single donors with incorporation of molecular oxygen, incorporation of two atoms of oxygen	
GO:0003707	GO Molecular Functions	steroid hormone receptor activity	5
GO:0004879	GO Molecular Functions	RNA polymerase II transcription factor activity, ligand-activated sequence-specific DNA binding	6
GO:0042802	GO Molecular Functions	identical protein binding	21
GO:0030235	GO Molecular Functions	nitric-oxide synthase regulator activity	4
GO:0004672	GO Molecular Functions	protein kinase activity	10
GO:0005524	GO Molecular Functions	ATP binding	18
GO:0002020	GO Molecular Functions	protease binding	6

Table S4. KEGG pathway enrichment analysis

ID	Description	Count
hsa05200	Pathways in cancer	24
hsa05207	Chemical carcinogenesis - receptor activation	15
hsa04726	Serotonergic synapse	11
hsa05160	Hepatitis C	10
hsa05161	Hepatitis B	10
hsa05215	Prostate cancer	8
hsa01522	Endocrine resistance	8
hsa04913	Ovarian steroidogenesis	6
hsa05417	Lipid and atherosclerosis	10
hsa05163	Human cytomegalovirus infection	10
hsa04923	Regulation of lipolysis in adipocytes	6
hsa04657	IL-17 signaling pathway	7
hsa04151	PI3K-Akt signaling pathway	12
hsa04915	Estrogen signaling pathway	8
hsa05162	Measles	8
hsa00590	Arachidonic acid metabolism	6
hsa04914	Progesterone-mediated oocyte maturation	7
hsa05145	Toxoplasmosis	7
hsa05165	Human papillomavirus infection	11
hsa04024	cAMP signaling pathway	9

References

1. Ma, Q.-H.; Shi, X.-F.; Fan, B.; Liu, D.-Y. Study on the chemical constituents from *Patrinia scabra. Zhong Yao Cai* **2012**, *35*, 1257–1259.
2. El-Seedi, H.R. Antimicrobial arylcoumarins from *Asphodelus microcarpus*. *J. Nat. Prod.* **2007**, *70*, 118–120.
3. Yang, Y. Study on chemical constituents of Polygonum capitatum Buch.-Ham. ex D. Don (I). *Acad. J. Nav. Med. Univ.* **2009**, *30*, 937–940.
4. Cai, L.M.; Huo, S.X.; Lin, J.; Wu, P.P. Chemical constituents of *Vernonia anthelmintica* (L.) Willd. *Chin. Tradit. Pat. Med.* **2012**, *34*, 2159–2161.
5. Jang, D.S.K.; Lee, J.M.; Kim, Y.M.; Kim, Y.S.; Kim, J.H.; Kim, J.S. Puerariafuran, a new inhibitor of advanced glycation end products (AGEs) isolated from the roots of *Pueraria lobata*. *Chem. Pharm. Bull.* **2006**, *54*, 1315–1317.
6. Fan, Y. Study on Chemical Constituents of the Roots of *Pueraria Lobata* (Willd.) Ohwi and Screening of Chinese Herbal Extracts with PTP1B Inhibitory. Master's Thesis, Jiangxi University of Chinese Medicine, Jiangxi, China, 2022.
7. Zhang, H.; Fu, X.; Cai, Y.; Li., D.; Zhang, J.; Zuo, A. Study on Chemical Constituents of Ethanol Extract from Euonymus amygdalifolius. *Chin. Pharm.* **2018**, *29*, 176–179.
8. Zhang, X.; Wang, M.; Peng, S.; Liu, F.Q.; Ding, L.S. Chemical Constituents of *Pueraria peduncularis. Chin. Tradit. Herb. Drugs* **2002**, *33*, 11–14.
9. Wang, X.L.; Yu, F.R.; Lin, L.B.; Xiao, J.; Zhang, Q.; Wang, L.; Duan, D.Z.; Xie, G. Constituents with potent alpha-glucosidase inhibitory activity from *Pueraria lobata* (Willd.) ohwi. *Bioorg. Med. Chem. Lett.* **2017**, *27*, 1993–1998.
10. Wang, Z.Y.; Wang, Z.Q.; Zhou, Y.B. Studies on the Chemical Constituents in Herb of *Caesalpinia sappan* L.(I). *Nat. Prod. Res. Dev.* 2010, 22, 590–593. https://doi.org/10.16333/j.1001-6880.2010.04.040.
11. Zhang, D.-W.; Ren, Y.-X.; Dai, S.-J.; Liu, W.; Li, G. Isoflavones from vines of *Pueraria lobata*. *China J. Chin. Mater. Medica* **2009**, *34*, 3217–3220.
12. Goto, H.; Terao, Y.; Akai, S. Synthesis of Various Kinds of Isoflavones, Isoflavanes, and BiphenylKetones and Their 1,1-Diphenyl-2-picrylhydrazyl Radical-Scavenging Activities. *Chem. Pharm. Bull.* **2009**, *54*, 346–360.
13. Zhu, C.C.; Deng, G.H.; Lin, C.C.; Study on chemical constituents of Picrasma quassioides [I]. Chinese Journal of Chinese Materia Medica, 2011, 36 (07): 886-890.
14. Wang, N.; Liu, J.S.; Wang, D.; Zhang, S.J.; Zhang, M. et al. Study on triterpenes of Euphorbia fischeriana. J. Qiqihar Univ. 2017, 33, 59–61.
15. Li, G.Q.; Li, Y.Y.; Tan, Z.J. Spectroscopic Identification of β-sitosterol. *Guangdong Chem. Ind.* 2018, *45*, 128+16.
16. Liu, F.; Dai, R.; Lü, F.; Meng, W.; Chen, Y.; Deng, Y. Isolation and Identification of Chemical Components from Total Phenolic Extracts of Dragon's Blood. *J. Beijing Inst. Technol.* **2014**, *34*, 102–105. https://doi.org/10.15918/j.tbit1001-0645.2014.01.012.
